# Assessing the Accuracy and Readability of Generative Artificial Intelligence Responses for Esophageal and Gastric Cancer Patients

**DOI:** 10.3390/jcm15082958

**Published:** 2026-04-13

**Authors:** Shanhu Ran, Wenlong Guan, Ran Wei, Yukun Chen, Bo Zhang, Yating Wang, Mingguang Zhang, Zixian Wang, Wei Liao, Fan Chen

**Affiliations:** 1 Department of Intensive Care Unit, Sun Yat-sen University Cancer Center, Guangzhou 510060, China; 2 State Key Laboratory of Oncology in South China, Guangdong Provincial Clinical Research Center for Cancer, Sun Yat-sen University, Guangzhou 510060, Chinawangzx@sysucc.org.cn (Z.W.); 3 Department of Medical Oncology, Sun Yat-sen University Cancer Center, Guangzhou 510060, China; 4 Department of Hepatobiliary and Pancreatic Surgery, Sun Yat-sen University Cancer Center, Guangzhou 510060, China; 5 Department of Medical Oncology, National Cancer Center/National Clinical Research Center for Cancer/Cancer Hospital, Chinese Academy of Medical Sciences and Peking Union Medical College, Beijing 100021, China; 6 Ascension All Saint Hospital, Racine, WI 53405, USA; 7 Department of Colorectal Surgery, National Cancer Center/National Clinical Research Center for Cancer/Cancer Hospital, Chinese Academy of Medical Sciences and Peking Union Medical College, Beijing 100021, China

**Keywords:** generative artificial intelligence, esophageal cancer, gastric cancer, accuracy, readability

## Abstract

**Background**: Generative artificial intelligence (GenAI) models are increasingly used for medical information retrieval, due to their accessibility and efficiency. However, the accuracy and readability of their responses, specifically for upper gastrointestinal cancers, remain inadequately evaluated. This gap highlights the need for rigorous assessment to ensure reliable patient education and clinical integration. **Objective**: This study aimed to assess the accuracy and readability of responses generated by four prominent GenAI models (Kimi, DeepSeek, ChatGPT, and Gemini) when addressing patient-focused questions related to esophageal and gastric cancers. **Methods**: Twenty-five standardized medical questions about esophageal and gastric cancer covering domains of disease definition, treatment and management were posed to each model. Responses were assessed by four oncologists for accuracy by a 5-point Likert scale and analyzed for readability using Flesch–Kincaid Reading Ease, Flesch–Kincaid Grade Level, and SMOG metrics. High-interest questions for patients were identified via questionnaires. **Results**: Comparing the accuracy of GenAI-generated responses, DeepSeek achieved the highest overall accuracy score and outperformed other models in questions about definitions and treatments, while ChatGPT excelled in management-related inquiries. In subgroup analysis, GenAI models exhibited higher accuracy in answering definition and management questions, which patients preferred to inquire, compared with questions about cancer therapies. The responses produced by all models required a reading capacity from 11th-grade to college level. **Conclusions**: This study revealed that in this comparative evaluation application of GenAI models, DeepSeek provides the most accurate responses for upper GI cancer inquiries about definition and treatment, while ChatGPT showed superiority in management-related questions. However, all models generate texts requiring advanced reading levels, highlighting a need for readability optimization without compromising accuracy. GenAI shows promise for patient education but requires rigorous validation for clinical integration.

## 1. Introduction

The rapid development of generative artificial intelligence (GenAI) models or large language models (LLMs) is enabling transformative applications within clinical practice, including diagnosis, medical case analysis, and drug discovery [1,2,3]. However, as GenAI deployment expands, patients and their families increasingly rely on these models for low-cost access to medical information and consultations. Ensuring the quality of content generated from patient–GenAI interactions has thus become imperative.

Prominent commercial generative AI models, including Kimi K2.0, DeepSeek-V3, ChatGPT-5, and Gemini 2.5 Pro, engage users in open-ended dialog. Responses are dynamically generated by integrating pretraining data, real-time online searches when knowledge gaps exist, user-typing context, and logical inference.

Some research indicates that text readability and the quality of core information critically determine whether patients can accurately comprehend online health information [4,5]. Consequently, rigorous assessments of both medical accuracy and readability of GenAI’s outputs are essential to direct the usage of GenAI in these scenarios. Previous studies have conducted evaluations of GenAI usage in clinical practice, but have rarely focused on upper gastrointestinal (GI) cancers [2,6]. Moreover, most of these studies have assessed only a limited set of clinical questions and failed to analyze subgroups of questions [7,8,9]. Consequently, there persists an unmet need for granularly evaluating LLM applications in upper GI cancers to enable clinical integration.

To address these shortcomings of the existing work, we assessed the accuracy and readability of responses from four commonly used GenAI models, Kimi K2.0, DeepSeek V3, ChatGPT-5, and Gemini 2.5 Pro, to patient questions about upper GI cancers. This study is a multi-model validation study, employing a cross-sectional design, and the ultimate goal is to assess the accuracy and readability of answering medical queries by generative AI models, in order to promote the effective use of GenAI in online medical consultations for esophageal and gastric cancer patients.

## 2. Materials and Methods

### 2.1. Question Setup

The questions were developed through a multi-step process. An initial item pool was generated from the literature and clinical practice guidelines. A multidisciplinary team including oncologists, surgeons, and clinical nurses then reviewed the items and selected the final 25 questions based on clinical relevance and coverage of the predefined domains, which contain definition, prevention, diagnosis, management, prognosis, and daily care [10,11,12]. Identical question sets (25 items each for esophageal and gastric cancer) were posed to four LLMs (Kimi K2.0, DeepSeek-V3, ChatGPT-5 and Gemini 2.5 Pro) in English in mid-September 2025. Responses were systematically recorded for subsequent analysis and a supervising author reviewed every AI output. Given substantial thematic overlap between esophageal and gastric cancers, parallel questions were designed for both diseases (Supplementary Data S1, Table S1a,b). All model interactions were conducted via their respective official web interfaces, and each question was asked in a separate clean session, using the default settings as provided by each platform. No additional system instructions or role-playing prompts were used, and the online search function was disabled for all models during testing. Responses were generated once per question; no responses were regenerated or selectively included (Supplementary Data S3).

Furthermore, we categorized the questions into three subgroups based on their content, which were “definition,” “treatment,” and “management” (Supplementary Data S1, Table S2; a unique code was assigned to each question). The definition subgroup comprised 5 questions addressing the definition and mechanisms related to upper gastrointestinal tumors. Answers to these questions are relatively fixed and widely accepted, often characterized as textbook-style. The treatment subgroup included 15 questions concerning disease diagnosis and treatment. Given the highly individualized nature of cancer treatment protocols, answers to these questions are typically not singular. The management subgroup consisted of 5 questions related to patient daily care and survival, which may be more closely aligned with the quality of life of patients. Such categorization allowed us to further analyze which types of questions LLMs could respond with greater accuracy.

To assess the accuracy of GenAI responses alongside patient preferences and cognitive factors, we conducted an exploratory questionnaire survey in patients with esophageal or gastric cancer. Eligible participants were adult patients diagnosed with esophageal or gastric cancer or their lineal relatives, recruited from the Department of Medical Oncology, Department of Thoracic Surgery, and Department of Gastric Surgery. All participants provided written informed consent and were able to understand and complete the questionnaire independently. A total of 34 questionnaires were distributed, of which 32 were completed (16 for esophageal cancer and 16 for gastric cancer), yielding a response rate of 94.1%. No sensitive personal information was collected.

### 2.2. Accuracy Assessment

All generated responses were independently evaluated by a panel of four physicians specialized in oncology (Supplementary Data S1, Tables S3 and S4). The raters were blinded to the GenAI models in order to mitigate potential bias. Responses were scored using a 5-point Likert scale [13,14,15]. Given the imbalanced distribution of questions across categories (treatment: 15 items; definition: 5 items; management: 5 items), the overall accuracy rate could be primarily driven by performance on treatment-related questions. To address this limitation in subgroup analyses, we conducted weighted calculations based on the accuracy rates of the three distinct question categories.

### 2.3. Inter-Rater Reliability

The inter-rater reliability was assessed by using intraclass correlation coefficients (ICCs) based on a two-way random effects model assuming consistency [16]. Before rating, all four experts took part in a training session using a set of responses that were not included in the main dataset. They were given a standardized scoring rubric, and each rater independently assigned scores. No consensus meetings were held, and the average score across the four raters was used for all subsequent analyses.

For esophageal cancer, the ICC for single measures was 0.325 (95% CI: 0.222–0.436; *p* < 0.01), and the ICC for average measures was 0.658 (95% CI: 0.557–0.768; *p* < 0.01). For gastric cancer, the ICC for single measures was 0.342 (95% CI: 0.239–0.453; *p* < 0.01), and the ICC for average measures was 0.675 (95% CI: 0.557–0.768; *p* < 0.01). These values indicate good reliability for the average of four raters [17].

### 2.4. Readability Assessment

Three validated readability metrics were employed (Supplementary Data S1, Tables S5 and S6). The Flesch–Kincaid Reading Ease (FKRE) Scale is scored between 0 and 100, and higher FKRE scores mean greater readability, and an FKRE score of 65 indicates standard comprehensibility. The Flesch–Kincaid Grade Level (FKGL) corresponds to the U.S. grade level required for comprehension [18], and a score ≤ 7.0 indicates readability for average adults. Simple Measure of Gobbledygook (SMOG) estimates the years of education needed for comprehension [19]. A lower score indicates superior readability for the FKGL and SMOG scales. We calculated all these scores on a professional readability analysis website [20].

### 2.5. Statistical Analysis

All statistical analyses were performed using R (version 4.4.1) and GraphPad Prism (version 9.5). Continuous variables were described using mean ± standard deviation (SD), and categorical variables as counts and percentages. The statistical approach for group comparisons was chosen based on a predefined, stepwise procedure to ensure robustness and control for error rates. The following decision framework was applied to all group comparisons. The normality of data distribution within each group was assessed using the Shapiro–Wilk test. The homogeneity of variances across groups was tested using Bartlett’s test. If data from all groups simultaneously passed both normality and homoscedasticity assumptions, a one-way Analysis of Variance was used. If the assumption of normality in any group or homoscedasticity was violated, the Kruskal–Wallis H test was used instead. Tukey’s Honest Significant Difference test was used for all pairwise comparisons following a significant ANOVA result, and 95% confidence intervals for mean differences were obtained from the Tukey test output. Dunn’s test with Bonferroni-adjusted *p*-values was applied for pairwise comparisons following a significant Kruskal–Wallis test result, and for descriptive purposes, 95% confidence intervals for median differences were additionally calculated using the Hodges–Lehmann estimation. A two-tailed *p*-value < 0.05 was considered statistically significant.

### 2.6. Sensitive Analysis

The selection of questions and the sample size was based primarily on feasibility considerations, rather than on a formal a priori power analysis. To evaluate the ability of the data to detect meaningful differences, we conducted a post hoc power analysis for each cancer type based on the primary analytical approach. For the esophageal cancer data, the statistical power exceeded 0.99, with Cohen’s f = 0.71, α = 0.05, 4 groups, and *n* = 25 per group. For the gastric cancer data, the overall comparison was highly significant (*p* < 0.001), and the effect size (ε^2^) was approximately 0.35. Given the large effect size, the study had adequate power to detect the existing differences.

In addition, we performed several sensitivity analyses to assess the robustness of our findings. When we varied the accuracy cutoff, the relative ranking of the models remained unchanged. For the accuracy analysis, we averaged the four experts’ scores for each model–question combination to reduce inter-rater variability. Given that this approach does not fully account for the repeated-measures structure, we conducted a sensitivity analysis using repeated-measures ANOVA for the esophageal cancer data and the Friedman test for the gastric cancer data. The main conclusions were robust across all analytical approaches.

## 3. Results

### 3.1. Accuracy Analysis

**Figure 1:**
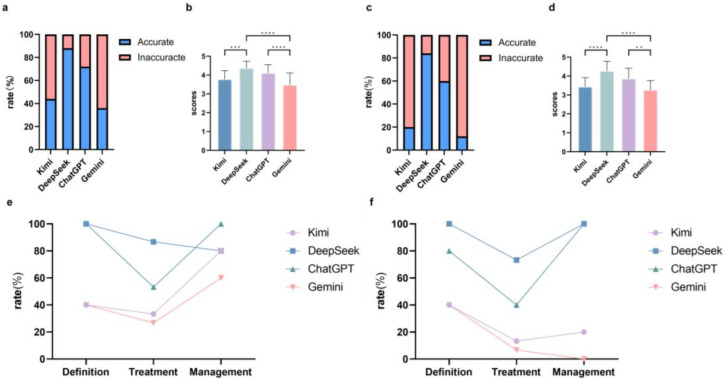
Accuracy analysis of GenAI responses to upper GI cancer-related questions. (**a**,**b**) Accuracy rate and accuracy scores of esophageal cancer responses. (**c**,**d**) Accuracy rate and accuracy scores of gastric cancer responses. (**e**,**f**) Accuracy rate of esophageal cancer and gastric cancer responses in subgroups, respectively. ** *p* < 0.01, *** *p* < 0.001, **** *p* < 0.0001.

For esophageal cancer-related questions, the accuracy rates on the responses were 44% (11/25) from Kimi, 88% (22/25) from DeepSeek, 72% (18/25) from ChatGPT, and 36% (9/25) from Gemini when responses with a mean score ≥ 4 were considered accurate. Given that the question set comprised an uneven distribution across clinical domains, we performed a weighted analysis, which showed that the accuracy rates were 51.1% from Kimi, 88.9% from DeepSeek, 84.4% from ChatGPT, and 42.2% from Gemini. Only DeepSeek (4.4 ± 0.72) and ChatGPT (4.1 ± 0.72) exceeded the overall accuracy threshold (>4.0). DeepSeek demonstrated significantly higher accuracy scores than Kimi (adjusted *p* < 0.001; 95%CI [0.23, 0.95]) and Gemini (adjusted *p* < 0.001; 95%CI [0.53, 1.25]) (Figure 1a,b; Table 1). ChatGPT also produced significantly more accurate answers than Gemini (adjusted *p* < 0.001; 95%CI [0.27, 0.97]). In the sensitive analysis using repeated-measures ANOVA, DeepSeek outperformed Kimi (adjusted *p* < 0.001; 95%CI [0.34, 0.84]) and Gemini (adjusted *p* < 0.001; 95%CI [0.62, 1.16]), and ChatGPT performed better than Gemini (adjusted *p* < 0.001; 95%CI [0.29, 0.97]), which was consistent with the original analysis.

Similarly for gastric cancer-related questions, accuracy rates were 20% (5/25) from Kimi, 84% (21/25) from DeepSeek, 60% (15/25) from ChatGPT, and 12% (3/25) from Gemini, respectively, and the weighted accuracy rates were 24.4% from Kimi, 91.1% from DeepSeek, 73.3% from ChatGPT, and 15.6% from Gemini. Although DeepSeek and ChatGPT both had more than 50% of replies that were deemed as accurate, only DeepSeek (4.3 ± 0.91) surpassed the overall accuracy threshold. DeepSeek significantly outperformed Kimi (adjusted *p* < 0.001; 95%CI [0.75, 1.25]) and Gemini (adjusted *p* < 0.001; 95%CI [0.75, 1.25]), while ChatGPT showed superior accuracy to Gemini (adjusted *p* = 0.005; 95%CI [0.25, 1.00]) (Figure 1c,d; Table 1). The sensitivity test revealed a significant overall difference among the four models (Friedman statistic = 43.87; df = 3, *p* < 0.001), consistent with the primary Kruskal–Wallis test.

These data suggested that the responses from DeepSeek and ChatGPT to medical questions associated with upper GI cancer were generally more precise and accurate than those of the other two GenAI models. The performance of DeepSeek was comparable to that of ChatGPT on questions for both cancer types, exhibiting no significant difference. To further test the stability of the descriptive findings, we performed sensitivity analyses using alternative thresholds of 3.5 and 4.5 (Supplementary Data S1, Table S7). In both cancer types, the ranking of models remained unchanged and these results confirm that the descriptive patterns are stable to reasonable variations in the accuracy definition.

### 3.2. Subgroup Analysis

To interrogate the performance of GenAI models in answering different categories of questions, we then analyzed the accuracy of the models responding to definition questions, treatment questions, and management questions, respectively. In esophageal cancer, there was no significant difference in scores between subgroups in Kimi (*p* = 0.335), DeepSeek (*p* = 0.825), ChatGPT (*p* = 0.072) and Gemini (*p* = 0.365), which implied that there was no significant difference when answering different types of questions within each GenAI model (Figure S1a–d). Both DeepSeek and ChatGPT demonstrated an accuracy of 100% in definition questions. DeepSeek achieved the highest accuracy rate (87%) in answering treatment questions, while ChatGPT performed the best in terms of accuracy (100%) in management questions (Figure 1e).

In gastric cancer, Gemini demonstrated a significantly higher accuracy in responding to definition questions than treatment questions (3.8 ± 0.47 vs. 3.1 ± 0.38, adjusted *p* = 0.034). No significant difference in accuracy was found among subgroups within Kimi (*p* = 0.694), DeepSeek (*p* = 0.079), and ChatGPT (*p* = 0.118) (Figure S1e–h). When comparing different GenAI models, DeepSeek manifested the highest accurate rate for all three categories, which was 100% for definition questions, 73% for treatment questions, and 100% for management questions. ChatGPT also achieved 100% accuracy for management questions (Figure 1f).

Together, these results suggested that generally DeepSeek outperformed other GenAI models in answering definition- and treatment-related questions, while ChatGPT was optimal for solving the questions specifically about management of upper GI cancers.

### 3.3. Patient Preference Questionnaire

**Figure 2:**
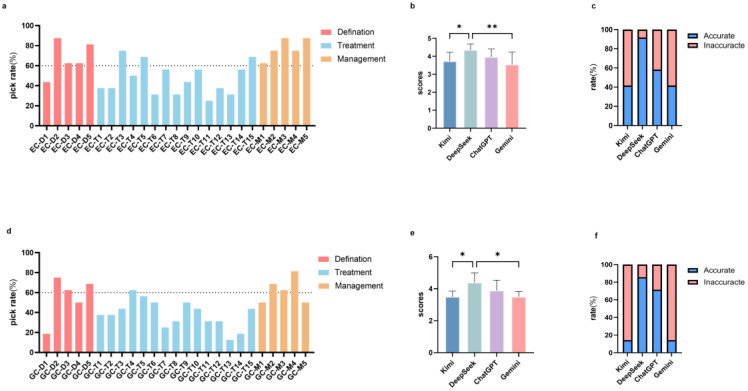
Accuracy analysis of GenAI responses to most selected questions. (**a**) Pick rate of esophageal questions in questionnaire. The dashed line indicates the 60% selection threshold. (**b**) Accuracy scores of the most picked (≥60%) esophageal cancer responses. (**c**) Accuracy rate of the most picked (≥60%) esophageal cancer responses. (**d**) Pick rate of gastric cancer questions in questionnaire. The dashed line indicates the 60% selection threshold. (**e**) Accuracy scores of the most picked (≥60%) gastric cancer responses. (**f**) Accuracy rate of the most picked (≥60%) gastric cancer responses. * *p* < 0.05, ** *p* < 0.01.

To better understand how these GenAI models performed in questions which patients were interested in and might ask frequently, we also conducted questionnaires on patients with gastric or esophageal cancers or their lineal family members. In esophageal cancer, the most frequently inquired questions, which were selected by 87.5% of survey respondents, included EC-D2 (What causes esophageal cancer?), EC-M3 (How to manage diet and exercise after having esophageal cancer?), and EC-M5 (Can esophageal cancer patients be cured, and is there a risk of recurrence after cure?). Another frequently asked question, selected by more than 80% of all participants, was EC-D5 (Whether esophageal cancer can be inherited, and whether relatives need testing?). Intriguingly, all management questions exhibited a 60% or higher selection frequency, and in general, definition and management questions exhibited stronger patient preferences than treatment questions on esophageal cancer (Figure 2a). For gastric cancer, the most frequently selected questions included GC-M4 (What should I pay attention to after discharge from gastric cancer surgery?) and GC-D2 (What causes gastric cancer?), selected by 81.25% and 75% of all participants respectively.

Then, to compare the performances of the four GenAI models in responding to the most frequently asked questions, we analyzed scores in questions selected by more than 60% of patients among different models (Table 2). The results showed that DeepSeek (4.4 ± 0.33) performed significantly better than Kimi (3.7 ± 0.49; adjusted *p* = 0.021; 95%CI [0.07, 1.18]) and Gemini (3.5 ± 0.69; adjusted *p* = 0.002; 95%CI [0.26, 1.37]) in esophageal cancer (Figure 2b,c).

Similarly, management questions demonstrated consistently higher inquiry rates by gastric cancer patients (Figure 2d). In gastric cancer, DeepSeek (4.4 ± 0.59) also exhibited higher accuracy scores than Kimi (3.5 ± 0.35; adjusted *p* = 0.013; 95%CI [0.16, 1.63]) and Gemini (3.5 ± 0.32; adjusted *p* = 0.013; 95%CI [0.16, 1.63]) (Figure 2e,f). Consistent with the overall accuracy analysis, DeepSeek showed superiority over the other two GenAI models, while it showed no statistically significant advantages over ChatGPT in either esophageal cancer (4.40 ± 0.33 vs. 4.00 ± 0.45; adjusted *p* = 0.238; 95%CI [−0.16, 0.95]) and gastric cancer (4.40 ± 0.59 vs. 3.90 ± 0.77; adjusted *p* = 0.264; 95%CI [−0.24, 1.24]).

### 3.4. Readability Analysis

**Figure 3:**
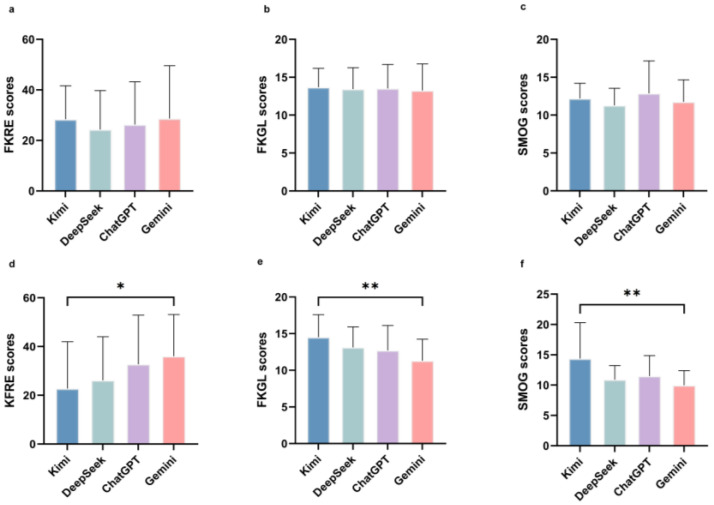
Readability analysis of GenAI responses to upper GI cancer-related questions. (**a**–**c**) Flesch–Kincaid Reading Ease scores, Flesch–Kincaid Grade Level scores and SMOG scores of esophageal cancer responses. (**d**–**f**) Flesch–Kincaid Reading Ease scores, Flesch–Kincaid Grade Level scores and SMOG scores of gastric cancer responses. * *p* < 0.05, ** *p* < 0.01.

In terms of esophageal cancer responses, all the four LLMs produced contents with mean FKRE scores below 30, which were categorized as very difficult. The average FKGL scores exceeded 13.0 among all models, indicating a college-level reading comprehension requirement. DeepSeek exhibited the lowest mean SMOG index (11.3 ± 2.3), yet its outputs remained challenging to comprehend. No significant inter-model differences were observed in FKRE (*p* = 0.781), FKGL (*p* = 0.968), and SMOG (*p* = 0.379) tests (Figure 3a–c; Table 3). According to FKRE scores in gastric cancer responses, Kimi and DeepSeek generated very difficult content, with an average score of less than 30. ChatGPT (32.7 ± 20.1) and Gemini (36.0 ± 17.1) scored within the range of being difficult, and Gemini’s FKRE score was significantly higher than Kimi’s (*p* = 0.044; [3.19, 24.10]). FKGL scores from all four GenAI models were above 11, which meant that they required an 11th-grade reading ability or higher. Gemini achieved the lowest FKGL index (11.3 ± 2.9), which was significantly lower than Kimi (14.5 ± 3.1; adjusted *p* = 0.002; 95%CI [−5.48, −0.96]). In terms of SMOG scores, Gemini (10.0 ± 2.4) presented a better readability than Kimi (14.4 ± 5.9; adjusted *p* = 0.003; 95%CI [−5.30, −1.50]) (Figure 3d–f; Table 3). The results in gastric cancer-related questions suggested that replies generated by Gemini may be the easiest to comprehend, while answers from Kimi required the highest education level to be understood by gastric cancer patients or relatives. 

## 4. Discussion

With the widespread adoption of the internet and rapid development of databases, most people have turned to online sources and browsers to seek health information [21]. As large language models emerge, their advantages, such as simplicity, efficiency, coherence, and personalization, have gradually positioned them as alternatives to traditional search engines [22]. The extensive use of GenAI models has influenced every aspect of life, including the retrieval of medical information. Therefore, the aim of this study was to assess the appropriateness and accuracy of exploring medical issues through GenAI models.

The results of this study revealed substantial variations in LLMs’ performance in responding to upper GI cancer-related inquiries. DeepSeek and ChatGPT demonstrated higher accuracy levels than the other two models. Uniquely, different GenAI models may excel at answering distinct categories of inquiries related to definitions, treatments, and managements of upper GI cancer. The accuracy scores of the responses to treatment inquiries were generally lower than those to definition and management response. Such discrepancy may arise from the fact that definitions and management protocols are relatively standardized and consistent, allowing LLMs to retrieve precise answers from pretrained data. However, treatment strategies were based on rapidly evolving evidence and personalized therapies and demanded real-time online searching.

Survey analysis indicates that patients and their family members preferred to consult definition and management information such as disease prognosis, self-care, and etiologic factors, the types of questions which GenAI models performed better in. This may be partially due to the fact that the responders were predominantly inpatients and had already received their treatments. Outpatients who have not started treatment may tend to consult the therapeutic options more frequently. Some studies are testing GenAI models for assisting clinicians with medical decisions [23,24,25], in order to potentially promote the rationality and accuracy of medical decision-making. Clinicians may ask more professional questions, or input detailed records of the medical history [26]. How LLMs perform in these inquiries needs to be assessed further.

As for readability, the calculation of readability scores relies on quantifiable linguistic features such as word/syllable counts, sentence length, and the proportion of complex words. The complexity of medical terminology, like seven syllables for esophageal cancer and four syllables for gastric cancer, may partly explain the respectively lower readability scores for esophageal cancer texts. In this study, the gastric cancer-related content generated by Gemini exhibited the highest readability, but its accuracy score was also the lowest, implying that its high readability may lead to a decline in accuracy. Therefore, optimizing readability without compromising accuracy remains an urgent need for future extensive use of GenAI models in such scenarios.

While the variabilities of GenAI model responses and the frequent updates of model versions may affect the long-term stability of our findings, our results reflect the performance of specific model versions at a specific time period. As models continue to evolve, the validity of these findings will need to be reassessed. Nevertheless, the fundamental differences in architectural paradigms, parameter organization strategies, training objectives, and task processing pathways across GenAI models persist across version updates. These underlying distinctions suggest that the relative performance patterns observed in this study may remain relevant, although direct confirmation would require future evaluations using updated model versions.

Our study focused specifically on upper gastrointestinal cancers, and the findings should be interpreted within this context. While some aspects of oncology care such as etiology, treatment principles, and supportive care share common elements across cancer types such as lung, colorectal, and nasopharyngeal cancers, and while patients and families often raise similar concerns, the generalizability of our findings to other tumor types cannot be assumed. Future studies that include different cancer types, languages, prompt formats, and question sets are needed to determine whether similar patterns hold. Therefore, the present study offers preliminary evidence for the validity and accuracy of GenAI models in addressing patient questions within the specific context of upper gastrointestinal cancers.

Furthermore, potential inherent biases may exist in AI-generated responses. First, models are trained on extensive historical datasets that may incorporate regional practice-pattern biases or omit the latest clinical trial evidence. Second, the algorithms might statistically prioritize recommendations with the highest frequency in databases or insights derived from the internal training database, instead of novel or tailored options. Third, the advice from GenAI may be biased if its training database does not adequately represent the diversity of information in this comparative evaluation of patient populations. Consequently, while these AI tools may generate broad summaries and overviews, their outputs must be interpreted with caution.

Based on this study, we have identified the advantages of certain GenAI models in answering medical questions raised by patients. Next, we plan to explore the impact of using AI models to assist in the self-management of patients after drug or surgical treatment on their quality of life, adverse reactions, and long-term treatment outcomes. Meanwhile, we have already embedded DeepSeek within the hospital information system in our institution and attempted to use it for analyzing patients’ medical histories and providing recommendations for potential treatment strategies. Prospective explorations of its accuracy will help us better apply GenAI models in the scenario of assisting doctors with medical decision-making.

Our study still has several limitations. The dividing our samples into multiple subgroups for analysis reduced the sample size in each group, which inherently increases the risk of Type I and II errors, although we employed rigorous post hoc corrections accordingly. In addition, the questionnaire was developed as an exploratory tool based on a literature review and multidisciplinary consensus, without formal quantitative inclusion or exclusion criteria. Demographic characteristics of participants were not collected, which limits the assessment of the sample’s representativeness and subgroup analyses.

When evaluating the context, we only rated one metric, which was accuracy, instead of separately assessing other dimensions such as comprehensiveness, timeliness, factual correctness, completeness, or clinical safety [27]. A more granular rubric would provide deeper insights. Otherwise, the readability analysis was conducted on English-language outputs, while the survey was administered to Chinese patients, limiting direct generalizability to non-English-speaking populations. Finally, as previously noted, although our findings may manifest substantial relevance across different cancer types, the extrapolation of these findings to non-oncological medical fields or clinical scenarios requires extreme caution.

## 5. Conclusions

To address medical questions raised by esophageal and gastric cancer patients, different GenAI models, namely Kimi, DeepSeek, ChatGPT, and Gemini, demonstrated distinct accuracy, among which DeepSeek achieved the highest accuracy. In the subgroup analysis, GenAI models exhibited higher accuracy in answering definition and management questions, which patients preferred to ask, compared with questions about cancer therapies. DeepSeek outperformed other models in addressing definition- and treatment-related questions; in contrast, ChatGPT demonstrated superiority specifically in answering questions about disease management. In general, GenAI models generated relatively difficult content in terms of readability when responding to inquiries associated with upper GI cancers. Further analyses are needed to explore whether GenAI models could optimize the quality of life, disease management, and potentially long-term benefits of esophageal and gastric cancer patients.

## Figures and Tables

**Table 1:** Accuracy scores of GenAI responses to upper GI cancer-related questions.

		Kimi	DeepSeek	ChatGPT	Gemini
Esophageal cancer	Overall mean (SD)	3.8 (0.77)	4.4 (0.72)	4.1 (0.72)	3.5 (0.92)
	Accuracy rate	11/25 (44%)	22/25 (88%)	18/25 (72%)	9/25 (36%)
	Equal-Weighted value	51.1%	88.9%	84.4%	42.2%
Gastric cancer	Overall mean (SD)	3.4 (0.80)	4.3 (0.91)	3.9 (0.77)	3.3 (0.79)
	Accuracy rate	5/25 (20%)	21/25 (84%)	15/25 (60%)	3/25 (12%)
	Equal-Weighted value	24.4%	91.1%	73.3%	15.6%

5-point Likert scale: 1 = Strongly disagree/Very inaccurate; 2 = Disagree/Inaccurate; 3 = Somewhat agree/Moderately accurate; 4 = Agree/Accurate; 5 = Strongly agree/Very accurate. Responses with mean scores ≥ 4.0 were deemed accurate. “Accuracy rate” refers to the proportion of responses with mean score ≥ 4.0. Equal-weighted value: arithmetic mean of accuracy rates of three categories of the question sets (Definition, Treatment and Management).

**Table 2:** Accuracy scores of GenAI responses to the most selected upper GI cancer questions.

		Kimi	DeepSeek	ChatGPT	Gemini
Esophageal cancer	Overall mean (SD)	3.7 (0.49)	4.4 (0.33)	4.0 (0.45)	3.5 (0.69)
	Accuracy rate	5/12 (42%)	11/12 (92%)	7/12 (58%)	5/12 (42%)
Gastric cancer	Overall mean (SD)	3.5 (0.35)	4.4 (0.59)	3.9 (0.64)	3.5 (0.32)
	Accuracy rate	1/7 (14%)	6/7 (86%)	5/7 (71%)	1/7 (14%)

5-point Likert scale: 1 = Strongly disagree/Very inaccurate; 2 = Disagree/Inaccurate; 3 = Somewhat agree/Moderately accurate; 4 = Agree/Accurate; 5 = Strongly agree/Very accurate. Responses with mean scores ≥ 4.0 were deemed accurate. “Accuracy rate” refers to the proportion of responses with mean score ≥ 4.0. Question selected by more than 60% of patients is deemed as most selected question.

**Table 3:** Readability scores of GenAI responses to upper GI cancer-related questions.

				DeepSeek			ChatGPT			Gemini		
	FKRE	FKGL	SMOG	FKRE	FKGL	SMOG	FKRE	FKGL	SMOG	FKRE	FKGL	SMOG
Overall mean (SD) of esophageal cancer	28.4 (13.2)	13.7 (2.5)	12.2 (2.0)	24.3 (15.4)	13.4 (2.8)	11.3 (2.3)	26.3 (16.9)	13.5 (3.2)	12.9 (4.3)	28.7 (20.9)	13.3 (3.5)	11.8 (2.9)
Overall mean (SD) of gastric cancer	22.8 (19.2)	14.5 (3.1)	14.4 (5.9)	26.1 (17.9)	13.1 (2.8)	10.9 (2.3)	32.7 (20.1)	12.7 (3.4)	11.5 (3.4)	36.0 (17.1)	11.3 (2.9)	10.0 (2.4)

Flesch Reading Ease score (FKRE) ranges from 1 to 100 (higher score is easier to read, and lower than 30 means very difficult). Flesch–Kincaid Grade Level (FKGL) ranges from1 to 18 (lower grade is easier to read; target benchmark: ≤7.0). SMOG formula estimates reading grade level (lower grade is easier to read).

## Data Availability

The raw data about the questionnaires are available under restricted access due to patient confidentiality and institutional agreements with the data custodian; access can be obtained by submitting a methodologically viable proposal to the corresponding authors via email. Other data generated or analyzed during this study are included in this published article and its Supplemental Information Files.

## Supplementary Materials

The following supporting information can be downloaded at https://www.mdpi.com/article/10.3390/jcm15082958/s1: The supplementary material includes Supplementary Table S1a: Question of esophageal cancer; Supplementary Table S1b: Question of gastric cancer; Supplementary Table S2: Classifications of esophageal cancer and gastric cancer related questions; Supplementary Table S3: Accuracy scores evaluation of GenAI model responses to esophageal cancer-related questions; Supplementary Table S4: Accuracy scores evaluation of GenAI model responses to gastric cancer-related questions; Supplementary Table S5: Readability scores of GenAI model responses to esophageal cancer-related questions; Supplementary Table S6: Readability scores of GenAI model responses to gastric cancer-related questions; Supplementary Table S7: Accuracy rate of esophageal cancer and gastric cancer using alternative thresholds of 3.5 and 4.5, respectively; Supplementary Figure S1: Accuracy Analysis of four AI models in subgroups of esophageal cancer and gastric cancer; Supplementary Data S3: questions and AI responses.
