# Supplementary material for: Assessing the Accuracy and Readability of Generative Artificial Intelligence Responses for Esophageal and Gastric Cancer Patients
Source: J Clin Med. 2026 Apr 13;15(8):2958. doi: 10.3390/jcm15082958 (PMC13116805; doi:10.3390/jcm15082958)
Supplement: Supplementary file 1 [file jcm-15-02958-s001.zip › Supplementary Data S1 JCM.pdf]

Assessing the Accuracy and Readability of Generative Artificial Intelligence Responses for Esophageal and Gastric Cancer Patients

Shanhu Ran [1,2,†], Wenlong Guan [2,3,†], Ran Wei [2,4,†], Yukun Chen [2,3,†], Bo Zhang [5], Yating Wang [6], Mingguang Zhang [7], Zixian Wang [2,3], Wei Liao [1,2,*] and Fan Chen [2,3,*]

1 Department of Intensive Care Unit, Sun Yat-sen University Cancer Center, Guangzhou 510060, China
2 State Key Laboratory of Oncology in South China, Guangdong Provincial Clinical Research Center for Cancer, Sun Yat-sen University, Guangzhou 510060, China; wangzx@sysucc.org.cn (Z.W.)
3 Department of Medical Oncology, Sun Yat-sen University Cancer Center, Guangzhou 510060, China
4 Department of Hepatobiliary and Pancreatic Surgery, Sun Yat-sen University Cancer Center, Guangzhou 510060, China
5 Department of Medical Oncology, National Cancer Center/National Clinical Research Center for Cancer/Cancer Hospital, Chinese Academy of Medical Sciences and Peking Union Medical College, Beijing 100021, China
6 Ascension All Saint Hospital, Racine, WI 53405, USA
7 Department of Colorectal Surgery, National Cancer Center/National Clinical Research Center for Cancer/Cancer Hospital, Chinese Academy of Medical Sciences and Peking Union Medical College, Beijing 100021, China
* Correspondence: liaowei@sysucc.org.cn (W.L.); chenfan2@sysucc.org.cn (F.C.)
† These authors contributed equally to this work.

Supplementary Table S1a. Question of esophageal cancer.

Q1	What is esophageal cancer?
Q2	What causes esophageal cancer?
Q3	How is esophageal cancer diagnosed?
Q4	How can I prevent esophageal cancer?
Q5	How to stage esophageal cancer?
Q6	What are the symptoms of esophageal cancer?
Q7	How long does esophageal cancer patients survive?
Q8	Whether esophageal cancer can be inherited, and whether relatives need testing?
Q9	How to manage diet and exercise after having esophageal cancer?
Q10	What are the treatment methods for early esophageal cancer?
Q11	What are the treatment methods for intermediate esophageal cancer?
Q12	What are the treatments methods for advanced esophageal cancer?
Q13	What are the surgical methods of esophageal cancer?
Q14	What should I pay attention to after discharge from esophageal cancer surgery?
Q15	What is the medication regimen for advanced esophageal cancer?
Q16	What is the principle of chemotherapy drug action in esophageal cancer?
Q17	What are the side effects of commonly used chemotherapy drugs for advanced esophageal cancer?
Q18	What are the options of immunotherapy for esophageal cancer?
Q19	What are the side effects of immunotherapy for esophageal cancer?
Q20	What are the targeted drugs for esophageal cancer treatment?
Q21	What are the side effects of targeted therapy for esophageal cancer?
Q22	Whether radiotherapy is necessary for esophageal cancer?
Q23	What are the side effects of radiotherapy for esophageal cancer?
Q24	How to evaluate the effectiveness of therapeutic regimens for esophageal cancer?
Q25	Can esophageal cancer patients be cured, and is there a risk of recurrence after cure?

Supplementary Table S1b. Question of gastric cancer.

Q1	What is gastric cancer?
Q2	What causes gastric cancer?
Q3	How is gastric cancer diagnosed?
Q4	How can I prevent gastric cancer?
Q5	How to stage gastric cancer?
Q6	What are the symptoms of gastric cancer?
Q7	How long does gastric cancer patients survive?
Q8	Whether gastric cancer can be inherited, and whether relatives need testing?
Q9	How to manage diet and exercise after having gastric cancer?
Q10	What are the treatment methods for early gastric cancer?
Q11	What are the treatment methods for intermediate gastric cancer?
Q12	What are the treatments methods for advanced gastric cancer?
Q13	What are the surgical methods of gastric cancer?
Q14	What should I pay attention to after discharge from gastric cancer surgery?
Q15	What is the medication regimen for advanced gastric cancer?
Q16	What is the principle of chemotherapy drug action in gastric cancer?
Q17	What are the side effects of commonly used chemotherapy drugs for advanced gastric cancer?
Q18	What are the options of immunotherapy for gastric cancer?
Q19	What are the side effects of immunotherapy for gastric cancer?
Q20	What are the targeted drugs for gastric cancer treatment?
Q21	What are the side effects of targeted therapy for gastric cancer?
Q22	Whether radiotherapy is necessary for gastric cancer?
Q23	What are the side effects of radiotherapy for gastric cancer?
Q24	How to evaluate the effectiveness of therapeutic regimens for gastric cancer?
Q25	Can gastric cancer patients be cured, and is there a risk of recurrence after cure?

Supplementary Table S2. Classifications of esophageal cancer and gastric cancer related questions. A unique code was assigned to each question. For example, EC-D1 refers to the first question in definition subgroup of esophageal cancer .GC-T1 refers to the first question in treatment subgroup of gastric cancer.

Definition	Treatment	Management
EC-D1:What is esophageal cancer?<br>EC-D2:What causes esophageal cancer?<br>EC-D3:How to stage esophageal cancer?<br>EC-D4:What are the symptoms of esophageal cancer?<br>EC-D5:Whether esophageal cancer can be inherited, and whether relatives need testing?	EC-T1:How is esophageal cancer diagnosed?<br>EC-T2:What are the treatment methods for early esophageal cancer?<br>EC-T3:What are the treatment methods for intermediate esophageal cancer?<br>EC-T4:What are the treatments methods for advanced esophageal cancer?<br>EC-T5:What are the surgical methods of esophageal cancer?<br>EC-T6:What is the medication regimen for advanced esophageal cancer?<br>EC-T7:What is the principle of chemotherapy drug action in esophageal cancer?<br>EC-T8:What are the side effects of commonly used chemotherapy drugs for advanced esophageal cancer?<br>EC-T9:What are the options of immunotherapy for esophageal cancer?<br>EC-T10:What are the side effects of immunotherapy for esophageal cancer?<br>EC-T11:What are the targeted drugs for esophageal cancer treatment?<br>EC-T12:What are the side effects of targeted therapy for esophageal cancer?<br>EC-T13:Whether radiotherapy is necessary for esophageal cancer?<br>EC-T14:What are the side effects of radiotherapy for esophageal cancer?<br>EC-T15:How to evaluate the effectiveness of therapeutic regimens for esophageal cancer?	EC-M1:How to prevent esophageal cancer?<br>EC-M2:How long is the survival time for patients with esophageal cancer?<br>EC-M3:How to manage diet and exercise after having esophageal cancer?<br>EC-M4:What should I pay attention to after discharge from esophageal cancer surgery?<br>EC-M5:Can esophageal cancer patients be cured, and is there a risk of recurrence after cure?
GC-D1:What is gastric cancer?<br>GC-D2:What causes gastric cancer?<br>GC-D3:How to stage gastric cancer?	GC-T1:How is gastric cancer diagnosed?<br>GC-T2:What are the treatment methods for early gastric cancer?<br>GC-T3:What are the treatment methods for intermediate gastric	GC-M1:How to prevent gastric cancer?<br>GC-M2:How long is the survival time for patients with gastric cancer?<br>GC-M3:How to manage diet and exercise after having gastric

	cancer?	cancer?
GC-D4:What are the symptoms of gastric cancer?	GC-T4:What are the treatments methods for advanced gastric cancer?	GC-M4:What should I pay attention to after discharge from gastric cancer surgery?
GC-D5:Whether gastric cancer can be inherited, and whether relatives need testing?	GC-T5:What are the surgical methods of gastric cancer?<br><br>GC-T6:What is the medication regimen for advanced gastric cancer?<br>GC-T7:What is the principle of chemotherapy drug action in gastric cancer?<br>GC-T8:What are the side effects of commonly used chemotherapy drugs for advanced gastric cancer?<br>GC-T9:What are the options of immunotherapy for gastric cancer?<br>GC-T10:What are the side effects of immunotherapy for gastric cancer?<br>GC-T11:What are the targeted drugs for gastric cancer treatment?<br>GC-T12:What are the side effects of targeted therapy for gastric cancer?<br>GC-T13:Whether radiotherapy is necessary for gastric cancer?<br>GC-T14:What are the side effects of radiotherapy for gastric cancer?<br><br>GC-T15:How to evaluate the effectiveness of therapeutic regimens for gastric cancer?	GC-M5:Can gastric cancer patients be cured, and is there a risk of recurrence after cure?

Supplementary Table S3. Accuracy scores evaluation of GenAI model responses to esophageal cancer-related questions. A, B, C and D refer to Kimi, DeepSeek, ChatGPT and Gemini, respectively. 5-point Likert scale: 1=Strongly disagree/Very inaccurate, 2=Disagree/Inaccurate, 3 =Somewhat agree/Moderately accurate, 4=Agree/Accurate, 5=Strongly agree/Very accurate.

	Yating Wang				Bo Zhang				Wenlong Guan				Fan Chen			
	A	B	C	D	A	B	C	D	A	B	C	D	A	B	C	D
Q1	3	5	4	3	4	5	5	4	3	4	5	3	3	3	4	3
Q2	3	5	4	3	4	4	5	5	5	5	5	5	4	5	5	4
Q3	3	5	5	5	3	4	4	5	3	4	5	4	5	4	4	4
Q4	3	5	4	3	4	5	5	5	3	5	5	5	3	4	4	3
Q5	3	5	4	3	3	3	5	4	4	4	4	3	4	4	3	2
Q6	3	5	3	3	5	5	5	5	5	5	5	5	5	4	4	4
Q7	3	5	4	3	5	4	5	3	5	3	5	3	4	3	3	2
Q8	3	5	3	3	4	4	4	4	4	4	5	4	3	4	4	3
Q9	4	5	4	3	5	4	5	5	5	5	5	5	4	4	4	4
Q10	4	5	5	3	5	5	3	4	5	4	3	3	4	3	4	3
Q11	4	5	3	4	4	5	4	4	4	5	3	4	3	5	3	4
Q12	3	5	4	3	3	4	5	3	4	4	4	4	4	4	4	4
Q13	4	5	4	4	3	3	3	3	4	4	5	2	3	4	4	2
Q14	5	5	5	5	4	5	5	4	4	5	5	4	3	4	4	3
Q15	4	5	5	5	4	4	4	4	3	5	4	3	3	3	4	2
Q16	4	5	5	4	5	4	5	3	4	5	4	3	3	4	4	2
Q17	4	5	5	4	5	4	5	4	4	4	4	4	3	4	4	3
Q18	4	5	3	3	3	3	4	3	2	3	4	2	4	4	4	3
Q19	4	5	5	3	3	5	5	4	4	5	4	2	4	5	4	2
Q20	4	5	4	3	3	3	3	3	1	2	2	1	3	5	4	2
Q21	4	5	3	3	4	4	4	3	3	3	3	2	3	4	4	2
Q22	4	5	4	3	4	5	4	5	4	4	3	4	3	4	3	4
Q23	4	5	4	4	4	5	4	3	4	4	4	4	4	4	3	3

Q24	3	5	3	4	5	5	5	5	5	5	4	4	4	4	4	3
Q25	4	5	4	4	5	5	4	4	4	4	4	4	4	4	4	3

Supplementary Table S4. Accuracy scores evaluation of GenAI model responses to gastric cancer-related questions. A, B, C and D refer to Kimi, DeepSeek, ChatGPT and Gemini, respectively. 5-point Likert scale: 1=Strongly disagree/Very inaccurate, 2=Disagree/Inaccurate, 3 =Somewhat agree/Moderately accurate, 4=Agree/Accurate, 5=Strongly agree/Very accurate.

	Yating Wang				Bo Zhang				Wenlong Guan				Fan Chen			
	A	B	C	D	A	B	C	D	A	B	C	D	A	B	C	D
Q1	4	5	4	4	5	4	4	5	4	5	5	4	3	4	4	4
Q2	3	5	4	3	4	5	5	5	4	5	5	3	3	5	4	3
Q3	3	5	4	5	4	4	5	5	2	5	5	3	3	4	4	3
Q4	3	5	4	3	4	5	5	4	4	5	5	4	3	4	4	4
Q5	4	5	3	4	3	3	3	4	4	5	3	3	4	5	3	3
Q6	3	5	5	4	5	4	4	3	5	5	4	3	3	4	4	3
Q7	3	5	5	1	4	5	5	3	2	5	4	3	3	4	4	2
Q8	3	5	4	3	3	5	5	5	3	4	4	4	2	4	3	5
Q9	3	5	4	3	4	5	4	4	5	5	4	4	4	4	4	4
Q10	3	5	4	3	4	5	5	5	5	2	4	4	3	4	3	3
Q11	3	5	3	3	3	3	3	4	1	2	2	3	4	2	3	2
Q12	3	5	3	3	3	5	4	4	2	5	3	3	4	3	2	3
Q13	4	5	4	3	3	4	4	3	4	4	4	3	4	3	3	3
Q14	4	5	4	4	3	3	5	4	3	4	5	2	2	4	3	3
Q15	4	5	4	3	4	3	3	4	2	2	4	3	4	4	3	2
Q16	4	5	3	3	5	5	4	3	5	5	4	3	4	4	3	2
Q17	4	5	4	3	4	4	3	3	5	5	4	3	4	4	3	3
Q18	3	5	4	3	3	4	3	4	3	4	3	2	4	5	4	3
Q19	3	5	5	3	3	4	4	3	3	4	5	2	3	4	4	3
Q20	3	5	3	3	3	3	3	3	3	1	4	2	3	5	4	3
Q21	3	5	5	3	4	4	4	3	3	4	4	2	3	4	4	3
Q22	4	5	4	4	3	3	3	3	2	2	2	2	4	3	3	3

Q23	3	5	3	3	3	4	5	4	4	4	5	4	3	3	4	3
Q24	3	5	3	2	5	5	4	4	3	5	5	4	4	4	4	3
Q25	3	5	3	3	3	4	4	4	4	4	5	3	3	4	4	3

Supplementary Table S5. Readability scores of GenAI model responses to esophageal cancer-related questions. Q1 refers to question1. FKRE, FKGL and SMOG refer to Flesch-Kincaid Reading Ease, Flesch-Kincaid Grade Level and Simple Measure of Gobbledygook, respectively*.

	Kimi			DeepSeek			ChatGPT			Gemini		
	FKRE	FKGL	SMOG	FKRE	FKGL	SMOG	FKRE	FKGL	SMOG	FKRE	FKGL	SMOG
Q1	33.5	13.1	12.0	33.2	15.1	11.9	35.8	12.4	10.5	41.6	10.9	10.8
Q2	19.6	14.4	12.3	36.9	11.7	10.4	28.9	12.5	10.3	29.3	12.4	10.1
Q3	28.9	14.5	12.4	38.3	11.9	12.0	37.5	11.7	11.9	50.8	9.2	9.4
Q4	42.2	10.9	10.3	38.0	11.7	10.6	54.2	8.4	9.7	67.2	7.0	6.6
Q5	57.3	9.3	8.3	56.3	7.5	6.8	56.9	8.2	8.1	64.8	7.5	7.0
Q6	41.4	9.5	8.0	42.8	9.9	8.9	40.2	9.3	7.5	47.2	11.9	11.1
Q7	41.3	12.0	11.1	39.3	11.1	10.1	64.4	7.6	8.7	26.7	14.6	12.5
Q8	10.7	18.0	15.2	16.6	13.9	12.3	24.4	12.5	11.1	8.0	15.2	11.6
Q9	47.3	10.3	9.8	46.2	9.9	9.2	36.2	10.8	9.6	46.8	10.0	10.3
Q10	23.6	14.1	12.9	13.4	17.2	15.4	19.7	15.8	14.7	1.0	17.3	15.2
Q11	22.3	14.7	13.2	1.0	19.0	16.3	21.3	16.5	14.1	1.0	16.3	12.0
Q12	39.0	12.0	11.2	16.0	13.8	11.3	22.0	15.1	12.9	28.3	12.4	10.8
Q13	31.3	12.7	11.6	15.9	14.6	12.8	24.6	15.5	14.3	1.0	17.2	12.7
Q14	19.9	14.8	13.9	37.2	11.2	10.3	31.2	13.5	12.7	42.3	9.9	9.3
Q15	9.7	16.5	13.8	7.8	15.0	11.7	13.6	13.9	11.0	10.9	17.1	14.4
Q16	14.7	16.5	15.2	28.6	13.4	12.9	31.9	13.3	12.4	29.5	12.6	13.2
Q17	17.1	15.7	12.9	8.3	15.6	12.5	1.0	18.0	19.0	1.0	18.0	20.7
Q18	26.9	13.4	11.3	12.3	16.6	13.4	11.9	15.7	12.9	11.3	16.2	12.9
Q19	9.3	16.1	12.9	9.4	14.8	10.8	1.0	18.0	25.0	17.3	15.8	12.9
Q20	12.6	15.8	14.4	10.5	14.7	11.7	16.5	14.1	12.3	39.6	10.7	10.1
Q21	26.1	14.0	12.9	17.3	13.0	9.5	3.8	18.0	17.2	1.0	18.0	14.1
Q22	23.9	15.0	14.1	1.0	19.1	14.6	16.6	15.7	13.2	15.0	18.0	15.2
Q23	30.4	16.6	14.5	37.8	11.3	9.4	1.0	18.0	22.9	49.2	10.1	9.6
Q24	50.3	9.0	8.9	16.0	12.2	7.8	32.2	10.8	8.8	37.8	13.4	11.6
Q25	30.7	13.3	11.9	28.3	11.9	9.7	30.4	12.7	11.1	48.0	9.9	9.8

*No preprocessing or cleaning was performed before calculating readability scores. The raw text generated by each model was entered directly into the online calculator without removing bullet points, headings, abbreviations, or hyphens. We have since verified that these formatting elements do not materially affect the readability scores.

Supplementary Table S6. Readability scores of GenAI model responses to gastric cancer-related questions. Q1 refers to question1. FKRE, FKGL and SMOG refer to Flesch-Kincaid Reading Ease, Flesch-Kincaid Grade Level and Simple Measure of Gobbledygook, respectively*.

	Kimi			DeepSeek			ChatGPT			Gemini		
	FKRE	FKGL	SMOG	FKRE	FKGL	SMOG	FKRE	FKGL	SMOG	FKRE	FKGL	SMOG
Q1	45.3	11.2	7.2	41.9	11.6	8.9	65.0	7.8	3.8	47.9	9.7	8.3
Q2	29.4	14.8	13.2	37.5	11.5	10.4	41.4	10.6	9.7	49.7	9.0	8.3
Q3	31.8	14.1	12.0	39.2	11.9	12.0	57.0	9.8	10.1	49.0	9.8	9.2
Q4	36.3	12.3	11.6	40.7	10.7	9.8	48.6	8.6	9.0	56.4	7.5	7.6
Q5	63.9	8.6	6.6	71.3	5.6	4.8	74.3	6.6	7.2	66.1	6.7	6.6
Q6	1.0	18.0	31.3	1.0	18.0	15.2	42.0	10.8	9.6	27.2	11.2	8.6
Q7	1.0	18.0	20.7	50.4	10.8	11.2	36.7	13.0	11.6	29.7	10.6	8.0
Q8	4.6	18.0	18.2	30.0	13.3	12.4	31.2	13.3	12.0	54.9	9.6	11.1
Q9	1.0	18.0	22.9	39.3	11.3	11.0	47.2	10.4	9.4	58.0	6.8	6.0
Q10	16.1	15.0	13.7	27.6	13.6	12.1	1.0	17.1	14.1	31.5	11.0	8.8
Q11	7.4	16.0	13.7	22.5	14.1	12.9	25.3	14.5	12.9	19.0	13.2	10.6
Q12	31.7	12.4	10.8	25.4	12.5	10.4	39.2	10.8	8.9	39.6	11.2	10.7
Q13	33.6	12.9	11.2	49.5	9.4	9.2	42.8	11.1	10.8	37.9	11.3	11.0
Q14	33.1	11.6	10.4	34.3	10.5	9.1	37.8	11.5	10.3	49.0	8.7	8.3
Q15	1.3	16.7	12.7	1.0	16.2	10.2	1.0	18.0	15.0	1.5	15.8	11.9
Q16	5.5	16.4	13.4	11.8	13.7	10.5	37.1	12.7	11.2	52.3	10.4	11.1
Q17	25.6	12.6	11.1	8.5	13.1	7.4	36.7	12.2	11.4	40.1	9.7	8.3
Q18	4.1	18.0	18.2	3.8	16.7	12.7	19.4	15.2	13.4	26.2	12.9	11.1
Q19	1.0	18.0	19.9	10.0	14.4	10.4	20.9	13.9	10.8	24.5	11.5	7.6
Q20	29.1	15.7	13.7	11.0	14.4	11.6	14.3	15.9	13.9	10.6	15.9	14.5
Q21	1.0	18.0	25.6	15.1	12.3	7.7	41.5	10.7	9.7	38.5	11.4	11.6
Q22	29.2	15.4	12.5	14.7	16.8	13.6	10.0	18.0	15.2	14.8	16.0	12.9
Q23	37.9	12.0	10.7	22.4	17.1	14.1	1.0	18.0	18.2	18.4	18.0	16.3
Q24	60.3	8.2	8.0	7.9	16.0	13.4	1.0	18.0	19.9	12.5	14.5	11.3
Q25	38.0	11.3	10.1	36.6	12.8	11.6	46.3	9.1	8.9	45.3	10.3	9.5

*No preprocessing or cleaning was performed before calculating readability scores. The raw text generated by each model was entered directly into the online calculator without removing bullet points, headings, abbreviations, or hyphens. We have since verified that these formatting elements do not materially affect the readability scores.

Supplementary Table S7. Accuracy rate of esophageal cancer and gastric cancer using alternative thresholds of 3.5 and 4.5, respectively.

Cancer Type	Model	Accuracy Rate (≥3.5)	Accuracy Rate (≥4.5)
Esophageal	Kimi	84% (21/25)	12% (3/25)
	DeepSeek	100% (25/25)	48% (12/25)
	ChatGPT	92% (23/25)	36% (9/25)
	Gemini	64% (16/25)	4% (1/25)
Gastric	Kimi	48% (12/25)	4% (1/25)
	DeepSeek	96% (24/25)	52% (13/25)
	ChatGPT	92% (23/25)	20% (5/25)
	Gemini	36% (9/25)	0% (0/25)
