# Supplementary material for: Assessing the Accuracy and Readability of Generative Artificial Intelligence Responses for Esophageal and Gastric Cancer Patients
Source: J Clin Med. 2026 Apr 13;15(8):2958. doi: 10.3390/jcm15082958 (PMC13116805; doi:10.3390/jcm15082958)
Supplement: Supplementary file 1 [file jcm-15-02958-s001.zip › Supplementary Data S2 JCM.pdf]

Assessing the Accuracy and Readability of Generative Artificial Intelligence Responses for Esophageal and Gastric Cancer Patients

Shanhu Ran [1,2,†], Wenlong Guan [2,3,†], Ran Wei [2,4,†], Yukun Chen [2,3,†], Bo Zhang [5], Yating Wang [6], Mingguang Zhang [7], Zixian Wang [2,3], Wei Liao [1,2,*] and Fan Chen [2,3,*]

[1] Department of Intensive Care Unit, Sun Yat-sen University Cancer Center, Guangzhou 510060, China
[2] State Key Laboratory of Oncology in South China, Guangdong Provincial Clinical Research Center for Cancer, Sun Yat-sen University, Guangzhou 510060, China; wangzx@sysucc.org.cn (Z.W.)
[3] Department of Medical Oncology, Sun Yat-sen University Cancer Center, Guangzhou 510060, China
[4] Department of Hepatobiliary and Pancreatic Surgery, Sun Yat-sen University Cancer Center, Guangzhou 510060, China
[5] Department of Medical Oncology, National Cancer Center/National Clinical Research Center for Cancer/Cancer Hospital, Chinese Academy of Medical Sciences and Peking Union Medical College, Beijing 100021, China
[6] Ascension All Saint Hospital, Racine, WI 53405, USA
[7] Department of Colorectal Surgery, National Cancer Center/National Clinical Research Center for Cancer/Cancer Hospital, Chinese Academy of Medical Sciences and Peking Union Medical College, Beijing 100021, China
* Correspondence: liaowei@sysucc.org.cn (W.L.); chenfan2@sysucc.org.cn (F.C.)
† These authors contributed equally to this work.

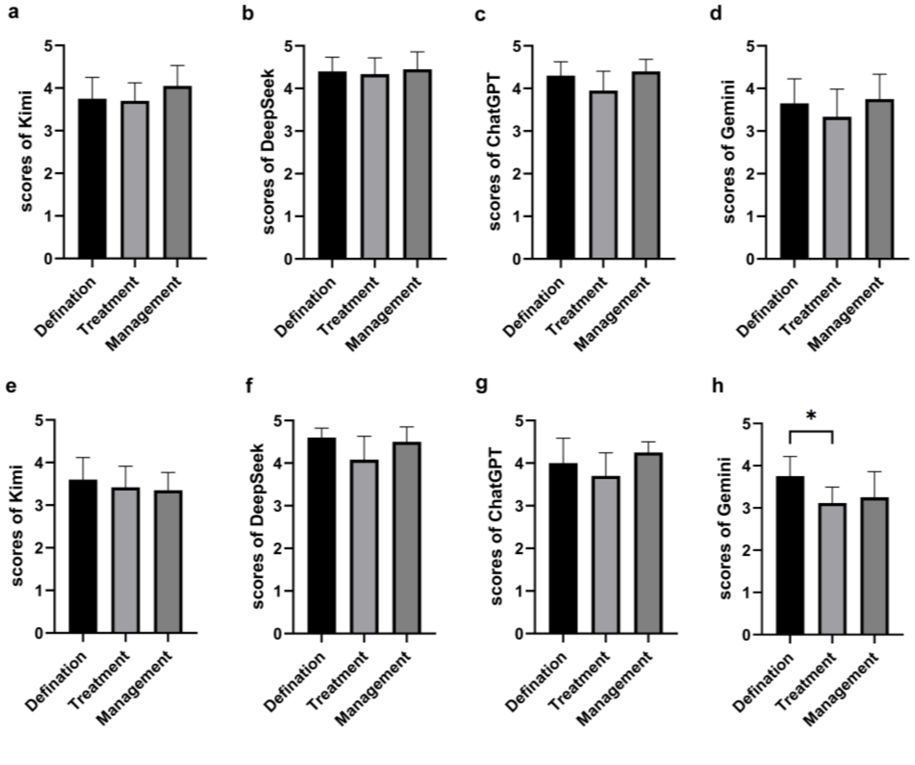

Suppl. Fig. S1│Accuracy Analysis of four AI models in subgroups of esophageal cancer and gastric cancer. a-d. Accuracy scores of esophageal cancer responses in subgroups, respectively. **e-f.** Accuracy scores of gastric cancer responses in subgroups, respectively.
