# Supplementary material for: Assessing the Accuracy and Readability of Generative Artificial Intelligence Responses for Esophageal and Gastric Cancer Patients
Source: J Clin Med. 2026 Apr 13;15(8):2958. doi: 10.3390/jcm15082958 (PMC13116805; doi:10.3390/jcm15082958)
Supplement: Supplementary file 1 [file jcm-15-02958-s001.zip › Supplementary Data S3 JCM.pdf]

Esophageal cancer questions and AI responses

All model interactions were conducted via their respective official web interfaces, and each question was asked in a separate clean session, using the default settings as provided by each platform. No additional system instructions or role-playing prompts were used, and the online search function was disabled for all models during testing. Responses were generated once per question; no responses were regenerated or selectively included.

Kimi

1.What is esophageal cancer
Esophageal cancer is a malignant tumor originating from the epithelial cells of the esophagus. It typically presents with symptoms such as difficulty swallowing, a feeling of obstruction or pain in the chest when swallowing, and weight loss. If it spreads or invades nearby organs, it can cause additional symptoms related to the affected areas.

2. What causes esophageal cancer?
Dietary factors： Consuming very hot foods and drinks can damage the esophageal lining. Eating pickled foods high in nitrites, which can convert into carcinogenic nitrosamines in the body, also increases the risk. Other factors include a diet low in fruits and vegetables, and high in processed meats.
Lifestyle factors：Smoking and excessive alcohol consumption are significant risk factors. Both can irritate the esophageal lining and increase the likelihood of cancerous changes.
Environmental factors： Exposure to certain chemicals and pollutants may contribute to the development of esophageal cancer.
Chronic conditions： Conditions such as gastroesophageal reflux disease (GERD) and Barrett's esophagus can increase the risk of developing esophageal cancer due to the constant irritation and damage to the esophageal lining.
Genetic factors： A family history of esophageal cancer may increase an individual's risk.

3.How is esophageal cancer diagnosed
Endoscopy：This is the primary diagnostic tool. It allows doctors to visually inspect the esophagus and take tissue samples (biopsies) for pathological examination to confirm the presence of cancer.
Imaging tests： X-ray with barium swallow, CT scans, MRI, and PET scans can help determine the size and location of the tumor, as well as whether it has spread to nearby lymph nodes or other organs.
Cytological tests：These include cytology brushings during endoscopy and balloon cytology, which can detect abnormal cells shed from the esophageal lining.
Tumor markers： Blood tests for certain tumor markers, such as CEA and CA199, can provide additional information but are not definitive for diagnosis.

4.How can I prevent esophageal cancer?

Healthy diet：Eat a diet rich in fruits and vegetables, and limit the intake of pickled and smoked foods. Avoid consuming very hot foods and drinks.

Lifestyle changes：Quit smoking and limit alcohol consumption. Maintain a healthy weight through regular exercise and a balanced diet.

Regular check-ups：If you have a family history of esophageal cancer or other risk factors, regular screenings can help detect any abnormalities early.

Manage chronic conditions：Treat and manage conditions like GERD to reduce the risk of developing Barrett's esophagus and esophageal cancer.

5.How to stage esophageal cancer?

Stage I：The cancer is confined to the inner layers of the esophagus and has not spread to lymph nodes or other organs. Patients may have mild symptoms or none at all.

Stage II：The cancer has grown into the muscle layer of the esophagus and may have spread to nearby lymph nodes but not to distant organs. Symptoms such as difficulty swallowing and chest pain may become more noticeable.

Stage III：The cancer has invaded the outer layers of the esophagus and may have spread to surrounding tissues and lymph nodes. Patients often experience significant difficulty swallowing, weight loss, and pain.

Stage IV：The cancer has spread to other parts of the body, such as the liver, lungs, or bones. This stage is associated with more severe symptoms and a poorer prognosis.

6.What are the symptoms of esophageal cancer?

The symptoms of esophageal cancer include：

Painful or difficult swallowing (dysphagia), which is the most prevalent symptom, reported in over 90% of patients.

Weight loss, experienced by approximately 50% of individuals with esophageal cancer.

Pain behind the breastbone.Hoarseness and cough.Indigestion and heartburn.A lump under the skin.

7.How long does esophageal cancer patients survive?

The survival time of esophageal cancer varies greatly depending on factors such as the stage at diagnosis, the patient's overall health, and the effectiveness of treatment. When esophageal cancer is found very early, there is a better chance of recovery. However, it is often diagnosed at an advanced stage, making it difficult to cure.

8.Whether esophageal cancer can be inherited and whether relatives need to be tested?

Esophageal cancer is not typically considered a strongly hereditary disease, but having a family history of esophageal cancer may increase the risk. Relatives of individuals with esophageal cancer may need to be tested, especially if they have other risk factors such as smoking, heavy alcohol use, or a history of gastroesophageal reflux disease.

9.How to manage diet and exercise after having esophageal cancer?

After getting esophageal cancer, managing diet and exercise is important. Patients should：

Adopt a balanced diet that is easy to swallow and digest, such as pureed foods or smoothies.
Eat small, frequent meals instead of large ones to reduce discomfort.
Avoid spicy, acidic, and hard foods that may irritate the esophagus.
Engage in regular, gentle exercise as tolerated, such as walking or yoga, to maintain overall health and strength.
Consult with a healthcare professional or dietitian to develop a personalized plan based on individual needs and treatment side effects.

10.What are the treatment methods for early esophageal cancer?
Early-stage esophageal cancer can be treated using:
Endoscopic Resection: This includes techniques such as Endoscopic Mucosal Resection (EMR) and Endoscopic Submucosal Dissection (ESD). These methods are minimally invasive and effective for superficial lesions with no lymph node involvement.
Surgery: For some early-stage cancers, surgery to remove the tumor and a small margin of normal tissue may be performed. This can be done through minimally invasive techniques.
Radiofrequency Ablation (RFA): This is another minimally invasive option that uses heat to destroy cancer cells.

11. What are the treatment methods for intermediate esophageal cancer?
For middle-stage esophageal cancer, the treatment options include:
Surgery: An esophagectomy (removal of part or all of the esophagus) may be performed. This can be combined with lymph node removal to check for metastasis.
Chemotherapy and Radiation Therapy: These are often used before surgery (neoadjuvant therapy) to shrink the tumor and make it easier to remove.
Combined Modality Therapy: A combination of surgery, chemotherapy, and radiation therapy may be used to improve outcomes.

12. What are the treatment methods for advanced esophageal cancer?
Advanced esophageal cancer is typically treated with:
Chemotherapy: This is often the primary treatment to control the growth of the cancer.
Radiation Therapy: This can be used to relieve symptoms such as pain or difficulty swallowing.
Palliative Care: Focuses on improving the quality of life for patients with advanced cancer.
Targeted Therapy and Immunotherapy: These newer treatments may be used in some cases to target specific cancer cells.

13. What are the surgical methods of esophageal cancer?
The main surgical methods for esophageal cancer include:
Esophagectomy: This involves removing part or all of the esophagus and reconnecting the remaining esophagus to the stomach. The extent of removal depends on the location and stage of the tumor.
Open Esophagectomy: This is performed through large incisions in the neck, chest, or abdomen.
Minimally Invasive Esophagectomy: This uses several small incisions and a laparoscope to remove the esophagus. It offers faster recovery and less blood loss.

Lymph Node Removal: Nearby lymph nodes are also removed during surgery to check for cancer spread.

14. What should I pay attention to after discharge from esophageal cancer surgery?
After discharge from the hospital, it is important to follow these guidelines:
Dietary Adjustments: Start with a liquid diet and gradually transition to soft foods. Avoid hard, dry, or spicy foods that may irritate the esophagus.
Follow-Up Appointments: Attend all follow-up appointments for monitoring and early detection of any recurrence.
Physical Activity: Engage in light physical activity as recommended by your healthcare provider to aid recovery.
Medication Adherence: Continue taking any prescribed medications, such as pain relievers or anti-reflux medications.
Monitor for Complications: Watch for signs of infection, difficulty swallowing, or other unusual symptoms and report them to your healthcare provider immediately.

15. What is the medication regimen for advanced esophageal cancer?
For advanced esophageal cancer, the treatment regimen often focuses on systemic therapy to control the disease. Common approaches include:
Chemotherapy: Regimens such as paclitaxel with carboplatin or fluorouracil with oxaliplatin are commonly used.
Immunotherapy: Drugs like nivolumab and pembrolizumab are used to target the PD-1/PD-L1 pathway, enhancing the immune system's ability to fight cancer.
Targeted Therapy: Monoclonal antibodies like ramucirumab and trastuzumab may be used to target specific pathways in cancer cells.

16. What is the principle of chemotherapy drug action in esophageal cancer?
Chemotherapy drugs work by:
Interfering with DNA Synthesis: Drugs like fluorouracil and capecitabine inhibit the synthesis of DNA, preventing cancer cells from dividing and growing.
Inducing Apoptosis: By disrupting the cell cycle, chemotherapy drugs cause cancer cells to undergo programmed cell death (apoptosis).
Targeting Rapidly Dividing Cells: Since cancer cells divide more rapidly than normal cells, chemotherapy drugs selectively target these fast-growing cells.

17. What are the side effects of commonly used chemotherapy drugs for advanced esophageal cancer?
Common chemotherapy drugs used for advanced esophageal cancer, such as fluorouracil, carboplatin, and paclitaxel, can cause several side effects:
Gastrointestinal Issues: Nausea, vomiting, diarrhea, and mouth sores are common.
Bone Marrow Suppression: This can lead to anemia, neutropenia (low white blood cell count), and thrombocytopenia (low platelet count).
Neurotoxicity: Drugs like oxaliplatin can cause peripheral neuropathy, leading to numbness or tingling in the hands and feet.

Cardiotoxicity: Some chemotherapy drugs may affect heart function, although this is less common with the drugs used for esophageal cancer.
Fatigue: General tiredness and weakness are frequent side effects of chemotherapy

18. What are the options of immunotherapy for esophageal cancer?
Immunotherapy has become an important treatment option for esophageal cancer, especially for advanced stages. The main options include:
PD-1/PD-L1 Inhibitors: These drugs target the PD-1/PD-L1 pathway to enhance the immune system's ability to attack cancer cells. Examples include nivolumab, pembrolizumab, and tislelizumab.
CTLA-4 Inhibitors: Drugs like ipilimumab can be used in combination with PD-1 inhibitors to further boost the immune response.
TIGIT Inhibitors: Emerging research is exploring the use of TIGIT inhibitors, which target another immune checkpoint receptor. These drugs may be used in combination with PD-1 inhibitors.

19. What are the side effects of immunotherapy for esophageal cancer?
Immunotherapy can cause several side effects, including:
Immune-Related Adverse Events: These can affect various organs, such as the skin (rash), gastrointestinal tract (diarrhea), liver (hepatitis), and endocrine system (thyroid dysfunction).
Fatigue: A common side effect of immunotherapy.
Inflammation: Inflammation of organs like the lungs (pneumonitis) or heart (myocarditis) can occur.
Autoimmune Reactions: The immune system may attack healthy tissues, leading to conditions like colitis or hypothyroidism.

20. What are the targeted drugs for esophageal cancer treatment?
Targeted therapy focuses on specific molecular targets in cancer cells. Key targeted drugs include:
Trastuzumab: This monoclonal antibody targets the HER2 receptor and is used in patients with HER2-positive esophageal cancer.
Ramucirumab: This drug targets the VEGF receptor and is used to inhibit angiogenesis, thereby reducing the blood supply to tumors.
Other Targeted Agents: Research is ongoing to identify new targets and develop additional targeted therapies for esophageal cancer.

21. What are the side effects of targeted therapy for esophageal cancer?
Targeted therapy can cause specific side effects depending on the drug used:
Trastuzumab: Side effects include cardiac toxicity, which can lead to heart failure in some cases.
Ramucirumab: Common side effects include hypertension, fatigue, and gastrointestinal perforation.
General Side Effects: Targeted therapies may also cause skin rashes, nausea, and changes in blood counts.

22. Whether radiotherapy is necessary for esophageal cancer?

Radiotherapy can be an essential part of the treatment plan for esophageal cancer, depending on the stage and specifics of the disease:
Neoadjuvant Therapy: Used before surgery to shrink the tumor and make it easier to remove.
Adjuvant Therapy: Given after surgery to eliminate any remaining cancer cells.
Palliative Therapy: Used to relieve symptoms in advanced cases, such as difficulty swallowing.

23.What are the side effects of radiotherapy for esophageal cancer?
Possible side effects of external radiation therapy for esophageal cancer can include skin changes in areas getting radiation, such as redness, blistering, and peeling; nausea and vomiting; diarrhea; fatigue; painful sores in the mouth and throat; dry mouth or thick saliva; and pain with swallowing. These side effects are often worse if chemotherapy is given at the same time as radiation. Most side effects of radiation are temporary, but some less common side effects can be permanent, such as a stricture (narrowing) in the esophagus or lung damage leading to breathing problems.

24. How to evaluate the effectiveness of therapeutic regimens for esophageal cancer?
Objective Response Rate (ORR): Measure the percentage of patients whose tumors shrink or disappear.
Progression-Free Survival (PFS): Track the time from treatment start to when the cancer progresses.
Overall Survival (OS): Monitor the time from treatment start to death.
Quality of Life (QoL): Assess the impact on daily life.
Side Effects (AEs): Observe any adverse reactions.
Biomarker Analysis: Test for specific markers to predict treatment response.

25.Can esophageal cancer patients be cured, and is there a risk of recurrence after cure?
Esophageal cancer can sometimes be cured with treatment, especially if it is detected early and treated aggressively. However, there is always a risk of recurrence, even after successful treatment. The recurrence rate can be influenced by factors such as the stage of the cancer at diagnosis, the type of treatment received, and the patient's overall health. For example, the recurrence rate associated with neoadjuvant chemoradiotherapy followed by surgery approaches 50%.

DeepSeek

1. What is esophageal cancer?
Esophageal cancer is a malignant tumor that develops in the esophagus, the muscular tube that connects the throat to the stomach and allows food and liquids to pass through. It typically originates in the inner lining of the esophagus and can spread to other parts of the body if not treated early. The two most common types are:
Adenocarcinoma: Often occurs in the lower part of the esophagus, near the stomach, and is associated with chronic acid reflux and Barrett's esophagus.
Squamous cell carcinoma: Typically occurs in the upper and middle parts of the esophagus and is

often linked to smoking and alcohol consumption.

3. What causes esophageal cancer?
The exact cause of esophageal cancer is not fully understood, but several risk factors have been identified:
Chronic acid reflux (GERD): Long-term reflux can lead to Barrett's esophagus, a precancerous condition.
Tobacco use: Smoking cigarettes, cigars, or using smokeless tobacco increases the risk.
Alcohol consumption: Heavy drinking, especially when combined with smoking, raises the risk.
Obesity: Excess body weight is linked to adenocarcinoma.
Diet: A diet low in fruits and vegetables or high in processed meats may increase risk.
Achalasia: A condition where the esophagus fails to move food to the stomach properly.
HPV infection: In some regions, human papillomavirus infection may contribute.
Age and gender: More common in people over 55 and in men.
Previous radiation therapy: Radiation to the chest or upper abdomen may increase risk.

3. How is esophageal cancer diagnosed?
Diagnosis typically involves a combination of tests:
Endoscopy: A thin, flexible tube with a camera (endoscope) is used to examine the esophagus and take tissue samples (biopsy).
Biopsy: Tissue samples are analyzed under a microscope to confirm cancer.
Imaging tests:
Barium swallow: The patient drinks a liquid containing barium, which coats the esophagus and makes it visible on X-rays.
CT scan: Provides detailed images of the esophagus and surrounding organs.
PET scan: Helps determine if cancer has spread.
Endoscopic ultrasound (EUS): Combines endoscopy and ultrasound to assess the depth of tumor invasion and lymph node involvement.
Lab tests: Blood tests may be used to assess overall health and organ function.

4. How can I prevent esophageal cancer?
While not all cases can be prevented, the following measures can reduce the risk:
Avoid tobacco: Quit smoking and avoid all forms of tobacco.
Limit alcohol: Drink in moderation or avoid alcohol altogether.
Maintain a healthy weight: Obesity is a risk factor, especially for adenocarcinoma.
Eat a balanced diet: Include plenty of fruits, vegetables, and whole grains.
Treat acid reflux: Manage GERD with medication or lifestyle changes to prevent Barrett's esophagus.
Regular check-ups: If you have Barrett's esophagus or other risk factors, regular monitoring can help detect precancerous changes early.

5. How to stage esophageal cancer?
Staging determines the extent of cancer spread and guides treatment. The TNM system is commonly used:

T (Tumor): Describes the size and depth of the primary tumor.
T1: Tumor invades the mucosa or submucosa.
T2: Tumor invades the muscularis propria.
T3: Tumor invades the outer layer of the esophagus.
T4: Tumor invades nearby structures (e.g., trachea, aorta).
N (Nodes): Indicates whether cancer has spread to lymph nodes.
N0: No lymph node involvement.
N1-N3: Increasing involvement of regional lymph nodes.
M (Metastasis): Indicates whether cancer has spread to distant organs.
M0: No distant metastasis.
M1: Distant metastasis present.
Stages:
Stage 0: Abnormal cells are present but have not invaded deeper tissues (carcinoma in situ).
Stage I: Cancer is limited to the inner layers of the esophagus.
Stage II: Cancer has spread to deeper layers or nearby lymph nodes.
Stage III: Cancer has invaded nearby structures or more lymph nodes.
Stage IV: Cancer has spread to distant organs (metastatic).

6. What are the symptoms of esophageal cancer?
Esophageal cancer often does not cause symptoms in its early stages. As the cancer grows, symptoms may include:
Difficulty swallowing (dysphagia): Feeling like food is stuck in the throat or chest.
Unintentional weight loss: Due to difficulty eating or loss of appetite.
Chest pain or discomfort: Often felt behind the breastbone or between the shoulder blades.
Chronic cough or hoarseness: Caused by irritation or nerve involvement.
Heartburn or indigestion: Persistent or worsening acid reflux.
Vomiting or regurgitation: Sometimes with blood.
Fatigue: Often due to anemia from chronic bleeding.
Black stools: A sign of gastrointestinal bleeding.
If these symptoms persist, it is important to seek medical evaluation.

7. How long does esophageal cancer patients survive?
Survival time depends on the stage at diagnosis, overall health, and treatment response. The 5-year survival rate (percentage of people alive 5 years after diagnosis) is:
Localized (early-stage): 47% (cancer is confined to the esophagus).
Regional spread: 25% (cancer has spread to nearby lymph nodes or tissues).
Distant metastasis: 5% (cancer has spread to distant organs).
Early detection and treatment significantly improve survival rates. However, esophageal cancer is often diagnosed at later stages, which reduces overall survival.

8. Whether esophageal cancer can be inherited and whether relatives need to be tested?
Inherited risk: Esophageal cancer itself is not directly inherited, but certain genetic conditions can increase susceptibility. For example:
Tylosis: A rare genetic disorder associated with a high risk of esophageal cancer.

Familial Barrett's esophagus: A family history of Barrett's esophagus may increase risk.
Lynch syndrome: A hereditary condition that increases the risk of several cancers, including esophageal cancer.
Testing relatives: Routine testing is not recommended unless there is a known genetic condition or strong family history of esophageal cancer or related conditions (e.g., Barrett's esophagus). In such cases, genetic counseling and testing may be considered.

9. How to manage diet and exercise after having esophageal cancer?
Managing diet and exercise is crucial for maintaining strength, improving treatment outcomes, and enhancing quality of life.
Dietary Management:
Small, frequent meals: Eat smaller portions more often to reduce strain on the esophagus.
Soft or liquid foods: Choose foods that are easy to swallow, such as soups, smoothies, and purees.
Avoid irritants: Spicy, acidic, or very hot foods can worsen symptoms.
High-calorie, high-protein foods: To prevent weight loss and maintain muscle mass (e.g., eggs, yogurt, nut butters).
Stay hydrated: Drink fluids throughout the day, but avoid large amounts at once.
Nutritional supplements: If eating is difficult, supplements or tube feeding may be necessary.
Work with a dietitian: To create a personalized meal plan that meets nutritional needs.
Exercise Management:
Light to moderate activity: Walking, stretching, or yoga can improve energy levels and reduce fatigue.
Strength training: Gentle resistance exercises can help maintain muscle mass.
Avoid overexertion: Listen to your body and rest when needed.
Consult your healthcare team: Before starting any exercise program, especially during or after treatment.
General Tips:
Manage side effects: Address treatment-related issues like nausea, fatigue, or pain with medical support.
Mental health: Seek counseling or support groups to cope with emotional challenges.
Follow-up care: Regular check-ups to monitor recovery and detect any recurrence early.

10. What are the treatment methods for early esophageal cancer?
Early esophageal cancer (localized to the mucosa or submucosa) can often be treated with curative intent. Treatment options include:
Endoscopic Resection: Techniques like endoscopic mucosal resection (EMR) or endoscopic submucosal dissection (ESD) are used to remove early-stage tumors confined to the superficial layers.
Surgery: Esophagectomy (removal of part or all of the esophagus) may be considered for early-stage cancer, especially if there is a risk of lymph node involvement.
Radiation Therapy: Sometimes used in combination with surgery or as a standalone treatment for early-stage cancer, particularly in patients who are not surgical candidates.
Chemotherapy: May be used in combination with radiation (chemoradiation) for certain

early-stage cancers to reduce the risk of recurrence.

11. What are the treatment methods for intermediate esophageal cancer?
Middle-stage esophageal cancer (locally advanced, involving deeper layers or regional lymph nodes) typically requires a multimodal approach:
Chemoradiation: A combination of chemotherapy and radiation therapy is often used as a primary treatment to shrink the tumor and improve resectability.
Surgery: Esophagectomy is commonly performed after chemoradiation if the tumor is deemed resectable.
Neoadjuvant Therapy: Chemotherapy or chemoradiation before surgery to reduce tumor size and improve outcomes.
Adjuvant Therapy: Additional chemotherapy or radiation after surgery to eliminate residual cancer cells and reduce the risk of recurrence.

12. What are the treatment methods for advanced esophageal cancer?
Advanced esophageal cancer (metastatic or unresectable) focuses on palliative care and symptom management, with the following approaches:
Chemotherapy: The primary treatment to control cancer growth and alleviate symptoms. Common regimens include platinum-based drugs (e.g., cisplatin) combined with other agents like fluorouracil (5-FU) or taxanes.
Immunotherapy: Checkpoint inhibitors (e.g., pembrolizumab, nivolumab) may be used for patients with specific biomarkers like PD-L1 expression.
Radiation Therapy: Used to relieve symptoms such as pain, dysphagia, or bleeding.
Targeted Therapy: Drugs like trastuzumab (for HER2-positive cancers) or ramucirumab may be used in specific cases.
Palliative Procedures: Stent placement, laser therapy, or photodynamic therapy to relieve obstruction and improve swallowing.
Supportive Care: Nutritional support, pain management, and psychological care to improve quality of life.

13. What are the surgical methods of esophageal cancer?
Surgical treatment for esophageal cancer depends on the tumor's location, stage, and patient's overall health. Common surgical methods include:
Esophagectomy: The primary surgical procedure, involving removal of part or all of the esophagus. Types include:
Transhiatal Esophagectomy: The esophagus is removed through incisions in the abdomen and neck, without opening the chest.
Transthoracic Esophagectomy: The esophagus is removed through incisions in the abdomen and chest (e.g., Ivor Lewis procedure).
Minimally Invasive Esophagectomy (MIE): Performed using laparoscopic or robotic techniques to reduce recovery time and complications.
Lymph Node Dissection: Removal of regional lymph nodes to assess and prevent cancer spread.
Reconstruction: After esophagectomy, the stomach is usually pulled up and connected to the remaining esophagus or a segment of the intestine is used to reconstruct the digestive tract

14. What should I pay attention to after discharge from esophageal cancer surgery?
Diet:
Start with a liquid or soft diet and gradually transition to solid foods as tolerated.
Eat small, frequent meals to avoid overloading the stomach.
Avoid spicy, acidic, or hard-to-digest foods.
Stay hydrated, but avoid drinking large amounts of fluids with meals.
Wound Care:
Keep the surgical incision clean and dry.
Watch for signs of infection (redness, swelling, discharge, fever).
Activity:
Avoid heavy lifting and strenuous activities for several weeks.
Gradually increase physical activity as tolerated.
Medications:
Take prescribed medications as directed, including PPIs, pain relievers, and any other medications.
Follow-Up Appointments:
Attend all scheduled follow-up appointments with your surgeon and oncologist.
Report any new or worsening symptoms, such as difficulty swallowing, pain, or weight loss.
Monitoring for Complications:
Be aware of signs of complications such as anastomotic leak (fever, chest pain, difficulty swallowing), infection, or blood clots.
Emotional Support:
Seek support from family, friends, or support groups to cope with the emotional and psychological impact of surgery and cancer.

15. What is the medication regimen for advanced esophageal cancer?
Chemotherapy:
Common regimens include:
Cisplatin + 5-Fluorouracil (5-FU): A standard combination.
Paclitaxel + Carboplatin: Often used in advanced or metastatic disease.
Docetaxel + Cisplatin + 5-FU (DCF): A more aggressive regimen.
Oxaliplatin + Capecitabine: An alternative to cisplatin-based regimens.
Targeted Therapy:
Trastuzumab: For HER2-positive esophageal adenocarcinoma.
Ramucirumab: An anti-angiogenic drug used in combination with paclitaxel for advanced disease.
Immunotherapy:
Pembrolizumab: For patients with PD-L1 positive tumors or MSI-H/dMMR tumors.
Nivolumab: Used in certain cases of advanced or metastatic esophageal cancer.
Palliative Care:
Medications to manage symptoms such as pain, nausea, and difficulty swallowing.

16. What is the principle of chemotherapy drug action in esophageal cancer?

Cisplatin:
Forms DNA cross-links, leading to DNA damage and inhibition of DNA replication and transcription, ultimately causing cancer cell death.
5-Fluorouracil (5-FU):
Inhibits thymidylate synthase, disrupting DNA synthesis and repair, leading to cell death.
Paclitaxel:
Stabilizes microtubules, preventing their disassembly, which interferes with cell division and leads to apoptosis.
Carboplatin:
Similar to cisplatin, it forms DNA cross-links, causing DNA damage and cell death.
Docetaxel:
Also a microtubule stabilizer, disrupting mitosis and leading to cancer cell death.
Oxaliplatin:
Forms DNA adducts, leading to DNA damage and cell death, with a different toxicity profile compared to cisplatin.

17. What are the side effects of commonly used chemotherapy drugs for advanced esophageal cancer?
Cisplatin:
Common Side Effects: Nausea, vomiting, kidney damage (nephrotoxicity), hearing loss (ototoxicity), peripheral neuropathy, low blood cell counts (myelosuppression).
Management: Hydration, antiemetics, monitoring of kidney function and hearing.
5-Fluorouracil (5-FU):
Common Side Effects: Diarrhea, mouth sores (mucositis), low blood cell counts, hand-foot syndrome (palmar-plantar erythrodysesthesia).
Management: Dose adjustments, supportive care for mucositis, and topical treatments for hand-foot syndrome.
Paclitaxel:
Common Side Effects: Peripheral neuropathy, allergic reactions, low blood cell counts, hair loss (alopecia).
Management: Premedication with steroids and antihistamines to prevent allergic reactions, dose adjustments for neuropathy.
Carboplatin:
Common Side Effects: Low blood cell counts, nausea, vomiting, kidney damage (less severe than cisplatin).
Management: Monitoring of blood counts and kidney function, antiemetics.
Docetaxel:
Common Side Effects: Low blood cell counts, fluid retention, peripheral neuropathy, hair loss.
Management: Premedication with steroids to reduce fluid retention, dose adjustments for neuropathy.
Oxaliplatin:
Common Side Effects: Peripheral neuropathy (often cold-induced), nausea, vomiting, low blood cell counts.
Management: Avoiding cold exposure, dose adjustments for neuropathy, antiemetics.

18. What are the options of immunotherapy for esophageal cancer?
Immunotherapy for esophageal cancer primarily involves the use of immune checkpoint inhibitors, which help the immune system recognize and attack cancer cells. The main options include:
PD-1/PD-L1 Inhibitors: These drugs block the PD-1/PD-L1 pathway, which cancer cells use to evade immune detection. Examples include:
Pembrolizumab (Keytruda): Approved for advanced esophageal squamous cell carcinoma (ESCC) and adenocarcinoma, particularly in patients with PD-L1 expression or high microsatellite instability (MSI-H).
Nivolumab (Opdivo): Approved for advanced esophageal squamous cell carcinoma, often as a second-line treatment.
Camrelizumab: Used in some regions for advanced esophageal squamous cell carcinoma.
CTLA-4 Inhibitors: Drugs like ipilimumab (Yervoy) target the CTLA-4 pathway, but they are less commonly used in esophageal cancer compared to PD-1/PD-L1 inhibitors.
Combination Therapies: Immunotherapy may be combined with chemotherapy, radiation, or other targeted therapies to enhance effectiveness.

19. What are the side effects of immunotherapy for esophageal cancer?
Immunotherapy can cause immune-related adverse events (irAEs) due to the overactivation of the immune system. Common side effects include:
Fatigue: A common side effect of many cancer treatments.
Skin Reactions: Rash, itching, or vitiligo (loss of skin pigment).
Gastrointestinal Issues: Diarrhea, colitis, or abdominal pain.
Hormonal Imbalances: Thyroid dysfunction, adrenal insufficiency, or hypophysitis (inflammation of the pituitary gland).
Liver Toxicity: Elevated liver enzymes or hepatitis.
Pneumonitis: Inflammation of the lungs, causing cough or shortness of breath.
Rare but Serious Side Effects: Myocarditis (heart inflammation), nephritis (kidney inflammation), or neurological issues.

20. What are the targeted drugs for the treatment of esophageal cancer?
Targeted therapy for esophageal cancer focuses on specific molecular targets involved in cancer growth and progression. Common targeted drugs include:
HER2 Inhibitors: For HER2-positive esophageal adenocarcinoma.
Trastuzumab (Herceptin): Often combined with chemotherapy.
Fam-trastuzumab deruxtecan (Enhertu): An antibody-drug conjugate used in HER2-positive cancers.
VEGF/VEGFR Inhibitors: Target blood vessel formation in tumors.
Ramucirumab (Cyramza): Used as a second-line treatment for advanced esophageal cancer, often in combination with chemotherapy.
EGFR Inhibitors: Less commonly used in esophageal cancer but may be considered in certain cases.
Cetuximab (Erbitux): Targets the EGFR pathway.

mTOR Inhibitors: Such as everolimus (Afinitor), used in some cases of advanced esophageal cancer.

21. What are the side effects of targeted therapy for esophageal cancer?
Targeted therapy can cause side effects depending on the specific drug and its mechanism of action. Common side effects include:
Skin Issues: Rash, dryness, or acne-like eruptions (common with EGFR inhibitors).
Hypertension: Often seen with VEGF/VEGFR inhibitors like ramucirumab.
Gastrointestinal Problems: Diarrhea, nausea, or vomiting.
Fatigue: A common side effect of many cancer treatments.
Infusion Reactions: Allergic reactions during drug administration (e.g., with trastuzumab).
Cardiac Toxicity: Heart-related issues, particularly with HER2 inhibitors like trastuzumab.
Bleeding or Blood Clotting Issues: Associated with VEGF/VEGFR inhibitors.
Liver or Kidney Dysfunction: Elevated liver enzymes or kidney impairment.

22. Whether radiotherapy is necessary for esophageal cancer?
Radiotherapy is often an important part of esophageal cancer treatment, but its necessity depends on several factors:
Stage of Cancer:
Early-Stage Cancer: Radiotherapy may be used as a primary treatment, often combined with chemotherapy (chemoradiation), especially for patients who are not surgical candidates.
Locally Advanced Cancer: Radiotherapy is commonly used alongside chemotherapy (neoadjuvant or definitive chemoradiation) to shrink tumors before surgery or as a standalone treatment.
Advanced/Metastatic Cancer: Radiotherapy may be used palliatively to relieve symptoms like pain or difficulty swallowing.
Type of Esophageal Cancer:
Squamous cell carcinoma and adenocarcinoma may respond differently to radiotherapy, with squamous cell carcinoma often being more radiosensitive.
Patient Health: Radiotherapy may not be suitable for patients with poor overall health or significant comorbidities.
Treatment Goals:
Curative Intent: Radiotherapy is often used in combination with other treatments to try to cure the cancer.
Palliative Intent: It can be used to improve quality of life by alleviating symptoms.

23. What are the side effects of radiotherapy for esophageal cancer?
Radiotherapy for esophageal cancer can cause both short-term (acute) and long-term (chronic) side effects. These depend on the radiation dose, treatment area, and individual patient factors. Common side effects include:
Short-Term Side Effects:
Fatigue: A common side effect during and after treatment.
Skin Reactions: Redness, irritation, or peeling of the skin in the treated area.
Esophagitis: Inflammation of the esophagus, causing pain, difficulty swallowing, or heartburn.
Nausea and Vomiting: Especially if the radiation field is near the stomach.

Loss of Appetite: Due to swallowing difficulties or nausea.
Weight Loss: Often resulting from reduced food intake.
Cough or Shortness of Breath: If the radiation field includes the lungs or airways.
Long-Term Side Effects:
Esophageal Stricture: Narrowing of the esophagus due to scarring, leading to swallowing difficulties.
Lung Damage: Radiation pneumonitis or fibrosis, causing chronic cough or breathing problems.
Heart Problems: Increased risk of pericarditis (inflammation of the heart lining) or cardiomyopathy if the heart is in the radiation field.
Secondary Cancers: Rarely, radiation can increase the risk of developing other cancers in the treated area.
Chronic Pain or Discomfort: In the chest or throat area.
Management of side effects may involve medications, dietary adjustments, or supportive care. Patients should report any persistent or severe symptoms to their healthcare team.

24. How to evaluate the effectiveness of therapeutic regimens for esophageal cancer?
The effectiveness of therapeutic regimens for esophageal cancer can be evaluated using several key metrics:
Patient Outcomes:
Survival rates (e.g., overall survival, progression-free survival).
Quality of life measures (e.g., symptom relief, functional status).
Treatment success rates (e.g., tumor response, recurrence rates).
Process Measures:
Adherence to clinical guidelines (e.g., use of recommended treatments like chemoradiation or surgery).
Timeliness of care (e.g., time from diagnosis to treatment initiation).
Multidisciplinary team involvement (e.g., coordination among oncologists, surgeons, and radiologists).
Patient Experience:
Patient satisfaction with care (e.g., communication, support, and accessibility).
Access to supportive services (e.g., nutrition counseling, pain management).
Safety and Complications:
Rates of treatment-related complications (e.g., infections, side effects).
Hospital readmission rates.
Cost-Effectiveness:
Resource utilization (e.g., hospital stays, medications).
Cost per quality-adjusted life year (QALY).
Research and Innovation:
Participation in clinical trials.
Adoption of new technologies or treatments.
Regular audits, patient feedback, and benchmarking against national or international standards are essential for continuous improvement.

25. Can esophageal cancer patients be cured, and is there a risk of recurrence after cure?

Early-Stage Cancer: Esophageal cancer detected at an early stage (localized to the esophagus) has a higher chance of being cured, especially with surgery, chemoradiation, or a combination of treatments.
Locally Advanced Cancer: Cure is possible but less likely. Multimodal treatment (surgery, chemotherapy, and radiation) offers the best chance.
Advanced/Metastatic Cancer: Cure is unlikely, but treatment can prolong survival and improve quality of life.
Risk of Recurrence:
Even after successful treatment, esophageal cancer has a significant risk of recurrence, particularly within the first 2-3 years. Factors influencing recurrence include:
Stage at diagnosis.
Type of treatment received.
Presence of residual cancer cells after treatment.
Patient health and lifestyle factors (e.g., smoking, alcohol use).
Regular follow-up is crucial to detect and manage recurrence early.

ChatGPT

1. What is esophageal cancer?
Esophageal cancer is a type of cancer that begins in the esophagus, the muscular tube connecting the throat to the stomach. It occurs when cells in the lining of the esophagus grow abnormally and uncontrollably. The two main types of esophageal cancer are:
Adenocarcinoma: Often begins in the lower part of the esophagus.
Squamous cell carcinoma: Typically occurs in the upper or middle part of the esophagus.

5. What causes esophageal cancer?
The exact cause of esophageal cancer is not always known, but certain factors increase the risk, including:
Chronic irritation of the esophagus from gastroesophageal reflux disease (GERD) or Barrett's esophagus.
Tobacco and alcohol use: These are major risk factors, especially for squamous cell carcinoma.
Obesity, which increases the risk of adenocarcinoma.
Dietary factors, such as a diet low in fruits and vegetables.
Achalasia, a condition where the esophagus has trouble moving food into the stomach.
Exposure to carcinogens, like certain chemicals.
Human papillomavirus (HPV), linked to squamous cell carcinoma in some cases.

3. How is esophageal cancer diagnosed?
Diagnosis typically involves:
Medical history and physical exam: Assessing symptoms like difficulty swallowing, weight loss, or chest pain.
Endoscopy: A flexible tube with a camera is used to examine the esophagus and take biopsies of suspicious areas.

Barium swallow: An X-ray test where the patient drinks a contrast liquid to highlight the esophagus.
Imaging tests: CT scans, PET scans, or MRIs to detect tumor spread.
Biopsy: Tissue samples are analyzed to confirm cancer.

4.How can I prevent esophageal cancer?
While not all cases can be prevented, risk can be reduced by:
Avoiding tobacco and limiting alcohol consumption.
Maintaining a healthy weight through diet and exercise.
Managing GERD to prevent Barrett's esophagus.
Eating a balanced diet rich in fruits, vegetables, and whole grains.
Regular screenings if you have high-risk conditions, such as Barrett’s esophagus.

5. How to stage esophageal cancer?
Staging helps determine the extent of cancer and guides treatment. It is based on the TNM system:
T (Tumor): How deep the tumor has grown into the esophageal wall.
N (Nodes): Whether cancer has spread to nearby lymph nodes.
M (Metastasis): Whether cancer has spread to distant organs.
Stages range from:
Stage 0 (carcinoma in situ): Abnormal cells in the innermost layer of the esophagus.
Stage I: Cancer is limited to the esophagus lining.
Stage II: Cancer has grown deeper and/or spread to nearby lymph nodes.
Stage III: Cancer has invaded surrounding tissues or more lymph nodes.
Stage IV: Cancer has metastasized to distant organs.
Imaging, endoscopic ultrasound, and biopsies are typically used to determine the stage.

6. What are the symptoms of esophageal cancer?
Symptoms often appear in later stages and may include:
Difficulty swallowing (dysphagia), often starting with solid foods and progressing to liquids.
Unexplained weight loss. Chest pain or discomfort, often behind the breastbone. Chronic cough or hoarseness. Vomiting or regurgitation of food. Persistent heartburn or indigestion. Fatigue.
Black, tarry stools (from bleeding in the esophagus).

7. How long does esophageal cancer patients survive?
Survival depends on the stage at diagnosis, the type of cancer, overall health, and treatment.
Early-stage (Stage I): The 5-year survival rate is around 47%.
Localized cancer: When confined to the esophagus, survival is higher.
Advanced stages (Stage IV): The 5-year survival rate is below 5%.
Median survival time for late-stage esophageal cancer may range from months to a few years with treatment.

8. Whether esophageal cancer can be inherited and whether relatives need to be tested?
Inheritance: Most cases are not hereditary. However, genetic predisposition may occur in families

with certain syndromes, such as Lynch syndrome or familial adenomatous polyposis (FAP).
Testing for Relatives: If there is a strong family history of esophageal cancer or related conditions, genetic counseling and testing may be advised. Relatives with symptoms or high-risk conditions (e.g., Barrett's esophagus) may benefit from regular screenings.

12. How to manage diet and exercise after having esophageal cancer?
Diet Management:
Soft, easily digestible foods: Avoid large or dry chunks that are hard to swallow.
Small, frequent meals: Prevents discomfort and ensures adequate nutrition.
High-calorie, nutrient-dense foods: Helps with weight maintenance (e.g., smoothies, soups, and pureed foods).
Avoid irritants: Limit spicy, acidic, or very hot foods and beverages.
Hydration: Drink plenty of fluids but avoid drinking with meals to prevent feeling full too quickly.
Dietitian support: A registered dietitian can create a personalized meal plan.
Exercise Management:
Light physical activity: Walking or gentle yoga can improve digestion and energy.
Avoid strenuous exercise: Particularly after surgery or during treatment.
Focus on strength: Gradually incorporate strength-building exercises as recovery progresses.

13. What are the treatment methods for early esophageal cancer?
Endoscopic therapies: Endoscopic mucosal resection (EMR) or endoscopic submucosal dissection (ESD) are used to remove early-stage tumors that are confined to the mucosal layer of the esophagus.
Radiofrequency ablation (RFA): This method uses heat to destroy cancerous cells in the early stage.
Chemotherapy or radiation: These can be used as adjuncts, especially if surgery or endoscopic procedures are not suitable.
Surgical options: In some cases, early-stage cancer may be treated with esophagectomy, which involves the surgical removal of part or all of the esophagus.

14. What are the treatment methods for intermediate esophageal cancer?
Surgical treatment: Esophagectomy, where part or the entire esophagus is removed, is the primary treatment for tumors that have spread into deeper layers but are still localized.
Chemoradiotherapy: A combination of chemotherapy and radiation therapy may be used either before surgery (neoadjuvant) to shrink the tumor or after surgery (adjuvant) to kill remaining cancer cells.
Endoscopic treatments: These may still be considered for tumors that are localized and have not spread beyond a certain area.

12. What are the treatment methods for advanced esophageal cancer?
Chemotherapy: Chemotherapy is often the main treatment for advanced esophageal cancer, especially if the cancer has spread to other parts of the body (metastasized). Drugs like cisplatin, paclitaxel, and 5-fluorouracil may be used.

Radiation therapy: This can help relieve symptoms like pain or difficulty swallowing and may be combined with chemotherapy (chemoradiation).
Targeted therapy: Drugs that target specific molecules involved in cancer growth, such as trastuzumab for HER2-positive esophageal cancer, may be used.
Immunotherapy: In certain cases, immune checkpoint inhibitors like pembrolizumab may be used to boost the immune system's ability to fight cancer.
Palliative care: In cases where the cancer cannot be treated effectively, focus may shift to alleviating symptoms and improving quality of life.

13. What are the surgical methods of esophageal cancer?
Esophagectomy: This is the most common surgical treatment, where part or all of the esophagus is removed. Depending on the location of the cancer, the surgery can be done through open surgery or minimally invasive (laparoscopic or robotic) techniques.
Esophagogastrectomy: This procedure involves removing part of the esophagus and part of the stomach, particularly when the cancer is near the junction between the two.
Palliative surgery: In advanced cases, surgery may be performed to bypass or relieve blockages in the esophagus to ease swallowing or provide a feeding route.
Endoscopic surgery: For early-stage cancers or in cases where open surgery is not suitable, minimally invasive endoscopic techniques can be used to resect small tumors.

14. What should I pay attention to after discharge from esophageal cancer surgery?
Dietary changes: Initially, you will need to follow a liquid or soft-food diet, gradually progressing to regular food as your esophagus heals. It's crucial to avoid eating large meals or foods that are difficult to swallow.
Monitoring for complications: Watch for signs of infection, such as fever, increased pain, redness, or swelling at the surgical site. Other signs of complications might include difficulty swallowing, chest pain, or signs of pneumonia.
Wound care: Follow instructions for caring for surgical wounds, including any drainage tubes or incisions.
Follow-up appointments: Regular check-ups with your oncologist and surgeon to monitor recovery and catch any signs of recurrence or complications early.
Physical activity: Gradual increase in physical activity is encouraged to promote healing, but avoid strenuous exercises until your doctor clears you.
Medications: Continue taking prescribed medications, such as pain relief, proton pump inhibitors, or antibiotics, as recommended by your doctor.

15. What is the medication regimen for advanced esophageal cancer?
Chemotherapy: Common regimens include:
Cisplatin-based therapy: Often combined with 5-fluorouracil (5-FU) or paclitaxel. This combination is widely used for advanced esophageal cancer.
Carboplatin with paclitaxel: Another combination for patients who may not tolerate cisplatin well.
Cisplatin with capecitabine: Sometimes used as an alternative to 5-FU.

Targeted therapy:
Trastuzumab: For HER2-positive cancers, trastuzumab (Herceptin) is used in combination with chemotherapy.
Ramucirumab: An anti-angiogenesis drug that blocks blood vessel formation in tumors, often combined with paclitaxel.
Immunotherapy:
Pembrolizumab (Keytruda) and nivolumab (Opdivo): These immune checkpoint inhibitors may be used in certain cases of advanced esophageal cancer, particularly if the cancer has high microsatellite instability (MSI-H) or PD-L1 expression.
Supportive medications: Pain relief, anti-nausea medications (e.g., ondansetron), and medications to manage side effects like diarrhea (e.g., loperamide) or constipation (e.g., stool softeners).

16. What is the principle of chemotherapy drug action in esophageal cancer?
Cisplatin and carboplatin: These are platinum-based chemotherapy drugs that work by damaging the DNA of cancer cells, preventing them from dividing and growing. They are highly effective against rapidly growing tumors.
5-Fluorouracil (5-FU): A chemotherapy drug that interferes with the production of thymidylate, a building block needed for DNA synthesis. This action stops cancer cells from growing and dividing.
Paclitaxel: Works by stabilizing microtubules, preventing them from breaking down during cell division, thus blocking the cancer cell’ s ability to divide and replicate.
Capecitabine: A prodrug that is converted into 5-FU inside the body, it inhibits DNA synthesis in cancer cells, ultimately causing cell death.
Trastuzumab: Targets HER2 receptors on cancer cells, inhibiting cell growth and promoting immune system attack on the cancer cells.

17. What are the side effects of commonly used chemotherapy drugs for advanced esophageal cancer?
Cisplatin:Nausea and vomiting, Kidney toxicity (nephrotoxicity), Hearing loss (ototoxicity)
Fatigue, Low blood counts (anemia, neutropenia, thrombocytopenia)
5-Fluorouracil (5-FU): Nausea and vomiting, Diarrhea, Mouth sores (stomatitis), Low blood cell counts, Hand-foot syndrome (redness, swelling, and pain on the palms of hands or soles of feet)
Paclitaxel: Hair loss (alopecia), Nausea and vomiting, Nerve damage (neuropathy), causing tingling, numbness, or pain in hands and feet, Low blood counts (leading to a higher risk of infection, bleeding, or fatigue), Fatigue
Capecitabine: Nausea and vomiting, Hand-foot syndrome, Diarrhea, Fatigue, Low blood counts
Trastuzumab: Heart problems (e.g., cardiomyopathy or heart failure), Infusion reactions (fever, chills, rash), Diarrhea
Ramucirumab: High blood pressure (hypertension), Fatigue, Diarrhea, Bleeding, Infections

18. What are the options of immunotherapy for esophageal cancer?
Immunotherapy options for esophageal cancer include:

Checkpoint inhibitors: These drugs block checkpoint proteins (such as PD-1 or PD-L1), allowing the immune system to recognize and attack cancer cells. Commonly used drugs include pembrolizumab (Keytruda), nivolumab (Opdivo), and atezolizumab (Tecentriq).
Monoclonal antibodies: These are engineered to target specific cancer cells. For esophageal cancer, the combination of nivolumab with chemotherapy is an option for advanced stages.

19. What are the side effects of immunotherapy for esophageal cancer?
The side effects of immunotherapy for esophageal cancer can include:
Fatigue
Skin reactions (rash, itching)
Diarrhea
Pneumonitis (lung inflammation)
Colitis (inflammation of the colon)
Endocrine disorders (hypothyroidism, adrenal insufficiency)
Liver inflammation (hepatitis)
Infusion-related reactions
Immunotherapy side effects can be severe in some cases, so careful monitoring is required.

20. What are the targeted drugs for the treatment of esophageal cancer?
Targeted drugs for esophageal cancer include:
Trastuzumab (Herceptin): Used for HER2-positive esophageal cancer.
Ramucirumab (Cyramza): An anti-VEGFR2 antibody used to inhibit blood vessel growth in tumors.
Lapatinib (Tykerb): A HER2/EGFR inhibitor, sometimes used in combination with other drugs.
Axitinib (Inlyta): A VEGF receptor inhibitor used in some cases.

21. What are the side effects of targeted therapy for esophageal cancer?
Side effects of targeted therapy for esophageal cancer can include:
Fatigue, High blood pressure, Diarrhea, Hand-foot syndrome (redness, swelling, or pain in the palms or soles), Liver toxicity, Blood clotting problems, Poor wound healing
These side effects are generally less severe than chemotherapy but still require monitoring during treatment.

22. Whether radiotherapy is necessary for esophageal cancer?
Radiotherapy may be necessary for esophageal cancer, depending on the stage and location of the tumor. It can be used in several ways:
Curative treatment: In combination with surgery or chemotherapy, radiation can help shrink the tumor and improve surgical outcomes.
Palliative care: For advanced stages, radiotherapy can relieve symptoms like difficulty swallowing or bleeding.
Adjuvant treatment: After surgery, radiotherapy can help eliminate any remaining cancer cells to reduce the risk of recurrence. The necessity and approach to radiotherapy are tailored to each patient, taking into account the tumor's characteristics, overall health, and treatment goals.

23. What are the side effects of radiotherapy for esophageal cancer?
Radiotherapy for esophageal cancer can cause various side effects, including: Fatigue, Skin irritation at the site of treatment, Difficulty swallowing (dysphagia), Sore throat, Nausea and vomiting, Loss of appetite, Diarrhea or constipation, Risk of lung or heart complications in the treated area, Dry mouth or mouth sores
These side effects vary based on the individual and the radiation dose.

24. How to evaluate the effectiveness of therapeutic regimens for esophageal cancer?
Imaging: CT, PET, or endoscopic ultrasound to assess tumor size and spread.
Symptoms: Improvement in swallowing or pain.
Biomarkers: Tumor markers like SCC or CEA.
Pathology: Biopsy for residual cancer after treatment.

25. Can esophageal cancer patients be cured, and is there a risk of recurrence after cure?
Esophageal cancer can be cured, particularly if diagnosed in early stages and treated effectively with surgery, radiation, and/or chemotherapy. However, there is always a risk of recurrence, even after successful treatment. The risk is higher in advanced stages, so long-term monitoring is crucial.

Gemini

1.What is esophageal cancer?
Esophageal cancer is the development of malignant (cancerous) cells in the tissues of the esophagus.
The esophagus is the muscular tube that connects your throat to your stomach.

6. What causes esophageal cancer?
Smoking: The most significant risk factor.
Heavy alcohol consumption: Increases the risk, especially when combined with smoking.
Gastroesophageal reflux disease (GERD): Chronic acid reflux can damage the lining of the esophagus, increasing the risk of adenocarcinoma.
Obesity: Linked to an increased risk of esophageal adenocarcinoma.
Barrett's esophagus: A condition where the normal cells lining the esophagus are replaced by abnormal cells, increasing the risk of adenocarcinoma.
Diet: A diet low in fruits and vegetables may increase the risk.
Other factors: Age (risk increases with age), family history of esophageal cancer.

3.How is esophageal cancer diagnosed?
Endoscopy: A thin, flexible tube with a light and camera is inserted through the mouth to examine the esophagus.
Biopsy: A small tissue sample is taken during endoscopy for examination under a microscope.
Imaging tests:
Chest X-ray: Can detect tumors.
CT scan: Provides detailed images of the chest and abdomen.

PET scan: Helps determine the extent of cancer spread.

4. How can I prevent esophageal cancer?
Quit smoking: This is the single most important step.
Limit alcohol consumption: If you drink, do so in moderation.
Maintain a healthy weight:
Eat a balanced diet: Include plenty of fruits, vegetables, and whole grains.
Manage GERD: If you have GERD, follow your doctor's treatment plan to control acid reflux.

5.How to stage esophageal cancer?
Staging describes the size and extent of the cancer, which helps determine the best treatment plan and prognosis. The most common staging system is the TNM system:
T (Tumor): Describes the size and location of the primary tumor.
N (Nodes): Indicates whether the cancer has spread to nearby lymph nodes.
M (Metastasis): Indicates whether the cancer has spread to distant parts of the body.

6.What are the symptoms of esophageal cancer?
Difficulty swallowing (dysphagia): This is the most common symptom and may start with trouble swallowing solid foods and progress to difficulty swallowing liquids.
Chest pain: May feel like heartburn, burning, or pressure.
Hoarseness:
Chronic cough
Unexplained weight loss
Vomiting
Coughing up blood
Important Note: Many of these symptoms can also be caused by other conditions, such as GERD.

7. How long does esophageal cancer patients survive?
Survival rates vary greatly depending on several factors, including:
Stage of cancer at diagnosis: Early-stage cancers have a better prognosis.
Type of cancer: Adenocarcinoma generally has a better prognosis than squamous cell carcinoma.
Overall health of the individual
Treatment options and their effectiveness
Five-year survival rates:
Early-stage: Can be over 90%
Late-stage: Can be below 20%

8.Whether esophageal cancer can be inherited and whether relatives need to be tested?
Esophageal cancer has a small hereditary component.
Certain genetic syndromes increase the risk of esophageal cancer, such as:
Familial adenomatous polyposis (FAP)
Lynch syndrome

9.How to manage diet and exercise after having esophageal cancer

Dietary changes:
Soft, easily digestible foods: Initially, you may need to consume soft foods like soups, purees, and mashed foods.
Small, frequent meals: Avoid large meals that can be difficult to swallow.
Avoid irritants: Limit or avoid acidic foods, spicy foods, alcohol, and caffeine, which can irritate the esophagus.
Maintain adequate nutrition: Ensure you're getting enough calories and nutrients. A registered dietitian can provide personalized guidance.
Exercise:
Regular physical activity: Can help improve overall health, manage weight, and reduce the risk of recurrence in some cases.

10.What are the treatment methods for early esophageal cancer?
Endoscopic mucosal resection (EMR) or endoscopic submucosal dissection (ESD): These minimally invasive procedures can be used to remove very small, early-stage tumors.
Esophagectomy: Surgical removal of the affected portion of the esophagus.

11.What are the treatment methods for intermediate esophageal cancer?
Esophagectomy: Often the primary treatment.
Neoadjuvant therapy: Chemotherapy and/or radiation therapy may be given before surgery to shrink the tumor and improve surgical outcomes.
Chemotherapy: May be used alone or in combination with radiation therapy.

12.What are the treatment methods for advanced esophageal cancer?
Chemotherapy and radiation therapy: May be used to control the cancer and relieve symptoms.
Palliative care: Focuses on managing symptoms and improving quality of life.

13.What are the surgical methods of esophageal cancer?
Esophagectomy:
Transthoracic esophagectomy: Access to the esophagus is through the chest.
Transhiatal esophagectomy: Access to the esophagus is through the abdomen.
Minimally invasive esophagectomy: Uses smaller incisions and advanced surgical techniques.
Esophagogastrostomy: The remaining portion of the esophagus is connected to the stomach.
Esophagojejunostomy: The remaining portion of the esophagus is connected to the jejunum (a part of the small intestine).

14. What should I pay attention to after discharge from esophageal cancer surgery?
Wound Care: Keep the incision clean and dry. Watch for signs of infection (redness, swelling, pus).
Diet:
Start with a liquid diet and gradually progress to soft foods.
Avoid foods that are irritating or difficult to swallow (spicy, acidic, hard-to-chew).
Eat small, frequent meals.
Swallowing Difficulties (Dysphagia):

Be patient as you relearn to swallow.
Consult a speech-language pathologist for swallowing exercises.
Pain Management:
Take pain medication as prescribed.
Notify your doctor if you experience severe or persistent pain.
Fatigue:
Rest when needed and avoid strenuous activity.
Side Effects of Treatment:
Monitor for side effects of chemotherapy or radiation (nausea, vomiting, diarrhea, mouth sores, etc.).
Contact your doctor immediately if you experience any concerning symptoms.
Follow-up Appointments:
Keep all scheduled follow-up appointments with your oncologist and other healthcare providers.
Emotional Support:
Seek support from family, friends, or support groups.
Consider counseling to help cope with the emotional and psychological impact of cancer.

15. What is the medication regimen for advanced esophageal cancer?
Chemotherapy:
First-line: Often includes a combination of drugs like:
Cisplatin and 5-FU，Paclitaxel and carboplatin，Docetaxel and cisplatin
Second-line: If the first-line treatment doesn't work or is no longer effective, other chemotherapy drugs may be used.
Immunotherapy:
Drugs like pembrolizumab and nivolumab may be used, especially in patients with specific genetic markers.
Targeted Therapy:
Drugs that target specific weaknesses in cancer cells may be used, such as trastuzumab for HER2-positive esophageal cancer.
Chemoradiotherapy: May be used in certain situations, especially for palliative purposes.

16.What is the principle of chemotherapy drug action in esophageal cancer?
Interfere with cell growth and division: Chemotherapy drugs work by targeting rapidly dividing cells, such as cancer cells. They interfere with various cellular processes, including:
DNA synthesis and repair: Preventing cancer cells from replicating their DNA.
Protein production: Disrupting the production of proteins essential for cell growth.
Cell division: Stopping cancer cells from dividing and multiplying.

17. What are the side effects of commonly used chemotherapy drugs for advanced esophageal cancer?
Cisplatin: Nausea and vomiting，Kidney damage，Hearing loss，Nerve damage
5-FU: Mouth sores，Diarrhea，Hand-foot syndrome (redness and pain in the palms and soles)
Paclitaxel: Nerve damage (numbness, tingling)，Hair loss，Muscle aches
Docetaxel: Fluid retention，Nail changes，Allergic reactions

Carboplatin: Kidney damage，Low blood platelet count (thrombocytopenia)

18. What are the options of immunotherapy for esophageal cancer?
Immune Checkpoint Inhibitors:
Pembrolizumab (Keytruda): Approved as a second-line treatment for esophageal squamous cell carcinoma.
Nivolumab (Opdivo): Approved as a second-line treatment for advanced esophageal squamous cell carcinoma and in combination with chemotherapy as a first-line treatment.
Nivolumab + Ipilimumab: Approved as a first-line treatment for advanced esophageal squamous cell carcinoma.

19. What are the side effects of immunotherapy for esophageal cancer?
Immune-related adverse events: These can include:
Fatigue
Skin rash
Pneumonitis (inflammation of the lungs)
Thyroid problems
Diarrhea
Liver problems

20. What are the targeted drugs for the treatment of esophageal cancer?
Trastuzumab (Herceptin): Used to treat HER2-positive esophageal cancer. HER2 is a protein that promotes the growth of some breast and other cancers.
Ramucirumab (Cyramza): May be used in combination with chemotherapy for advanced esophageal cancer.

21. What are the side effects of targeted therapy for esophageal cancer?
Trastuzumab:Heart problems，Liver problems，Infusion reactions
Ramucirumab:High blood pressure，Bleeding，Low platelet count

22. Whether radiotherapy is necessary for esophageal cancer?
Role of Radiotherapy:
Radiotherapy uses high-energy rays to kill cancer cells. It is often used in combination with chemotherapy to treat esophageal cancer.
It may be used before surgery to shrink the tumor, after surgery to destroy any remaining cancer cells, or as the primary treatment for patients who are not candidates for surgery.
Necessity:
The need for radiotherapy depends on several factors, including:
The stage and type of esophageal cancer
The overall health of the patient
The patient's treatment goals
The availability of treatment options

Decision-making:
The decision to use radiotherapy is made on an individual basis by a team of healthcare professionals, including an oncologist, radiation oncologist, and surgeon.

23. What are the Side effects of radiotherapy for esophageal cancer?
Common Side Effects:
Fatigue: Tiredness is a common side effect of radiation therapy.
Skin irritation: The skin in the treatment area may become red, sore, or dry.
Mouth sores: Radiation can cause sores and inflammation in the mouth and throat.
Difficulty swallowing (dysphagia): This can make eating and drinking uncomfortable.
Changes in taste: You may experience changes in your sense of taste.
Nausea and vomiting: These side effects may occur, although they are less common with modern radiation techniques.
Long-term Side Effects:
Esophageal stricture: Radiation can cause scarring in the esophagus, leading to narrowing and difficulty swallowing.
Lung damage: Radiation to the chest can sometimes damage lung tissue.
Heart problems: In some cases, radiation to the chest can increase the risk of heart disease.
Second cancers: There is a small increased risk of developing a second cancer years after radiation therapy.

24. How to evaluate the effectiveness of therapeutic regimens for esophageal cancer?
For esophageal cancer, the effectiveness of a treatment regimen is typically evaluated by considering factors such as:
Overall survival (OS): This is the percentage of patients who are still alive after a certain period of time, often five years, following treatment.
Progression-free survival (PFS): This is the length of time that patients live without their cancer worsening after treatment.
Disease-free survival (DFS): This is the length of time that patients live without any evidence of cancer after treatment.
Locoregional recurrence: This is the return of cancer in the same area where it was originally removed.
Distant metastasis: This is the spread of cancer to other parts of the body.
Treatment-related side effects: The severity and frequency of side effects caused by the treatment regimen are also considered.

25. Can esophageal cancer patients be cured, and is there a risk of recurrence after cure?
In some cases, esophageal cancer can be cured with treatment. The likelihood of cure depends on various factors, including the stage and type of cancer, the patient's overall health, and the effectiveness of the treatment.
Risk of Recurrence:
Even after successful treatment, there is always a risk of the cancer returning (recurrence).
Recurrence can occur locally in the esophagus or in other parts of the body.
Regular follow-up appointments are crucial for early detection of any recurrence.

Gastric cancer questions and AI responses

Kimi

1. What is gastric cancer?
gastric cancer, also known as gastric cancer, is a malignant tumor that originates from the cells lining the stomach. The most common type is adenocarcinoma, which accounts for over 95% of all gastric cancers.

2. What causes gastric cancer?
Dietary factors: A diet high in salted, pickled, or smoked foods and low in fresh fruits and vegetables is associated with an increased risk of gastric cancer.
Helicobacter pylori infection: This bacterium can cause chronic inflammation in the stomach, which may lead to cellular changes and increase the risk of developing gastric cancer.
Genetic factors: Having a family history of gastric cancer can increase an individual's risk due to genetic predisposition.
Precancerous conditions: Conditions such as chronic atrophic gastritis, gastric ulcers, and gastric polyps can increase the risk of gastric cancer.

3. How is gastric cancer diagnosed?
Endoscopy: A gastroscopy allows doctors to visually inspect the stomach lining and take biopsies for pathological examination.
Imaging tests: CT scans and MRI can help determine the size and location of the tumor and whether it has spread to other organs.
Laboratory tests: Blood tests may reveal anemia or other signs of cancer, and tumor markers can sometimes indicate the presence of gastric cancer.

4. How can I prevent gastric cancer?
Healthy diet: Eat a diet rich in fresh fruits and vegetables and limit the intake of salted, pickled, and smoked foods.
Regular exercise: Maintain a healthy lifestyle with regular physical activity.
Avoid smoking and limit alcohol: Both smoking and excessive alcohol consumption are risk factors for gastric cancer.
Screening: Individuals with a family history of gastric cancer or other high-risk factors should consider regular screening.

5. How to stage gastric cancer?
Staging is based on the TNM system:
T (Tumor): Describes the size of the original tumor and how far it has spread into nearby tissue.
N (Node): Indicates whether the cancer has spread to nearby lymph nodes.
M (Metastasis): Refers to whether the cancer has spread to other parts of the body.
Stages range from Stage 0 (carcinoma in situ) to Stage IV (advanced cancer with distant metastasis).

6. What are the symptoms of gastric cancer?
Early symptoms:
Upper abdominal discomfort or pain, often described as a feeling of fullness, dull ache, or mild pain, sometimes related to eating。
Digestive symptoms such as decreased appetite, early satiety, nausea, belching, and heartburn。
Mild weight loss without an obvious cause。
Black stool or vomiting blood in rare cases。
Advanced symptoms:
Progressive weight loss and cachexia。
Severe pain in the upper abdomen as the tumor grows or invades surrounding tissues。
Difficulty swallowing, especially with solid foods, if the cancer is located in the cardia。
Pyloric obstruction, causing nausea and vomiting, particularly of undigested food。
Increased bleeding, such as vomiting blood, black stool, or even acute massive bleeding。
Abdominal mass with accompanying bloating and ascites。
Metastasis symptoms, such as liver enlargement with jaundice, bone pain if metastasized to bones, or swollen, hard lymph nodes in the supraclavicular area。

7. How long does gastric cancer patients survive?
The survival time for gastric cancer depends on the stage of the cancer, the effectiveness of treatment, and the patient's overall health。
Stage 0: The five-year survival rate is approximately 89%。
Stage I: If the tumor is confined to the stomach, the survival rate is 78%; if it has spread to nearby lymph nodes, the survival rate may drop to 58%。
Stage II: The five-year survival rate is around 34%。
Stage IIIA: The survival rate is 20%。
Stage IIIB: The survival rate is 8%。
Stage IV: The five-year survival rate is about 7%。

8. Whether gastric cancer can be inherited, and whether relatives need testing?
Gastric cancer can have a genetic component. In about 5% to 10% of cases, gastric cancer is hereditary, with the most common type being hereditary diffuse gastric cancer, which is an autosomal dominant genetic disease。
Family members share common genetic factors, living environments, and dietary habits, which can lead to a familial clustering of gastric cancer。
Relatives of individuals with gastric cancer should consider regular screening, especially if there is a family history of the disease。

9. How to manage diet and exercise after having gastric cancer?
Diet management:
Consume a diet rich in fresh fruits and vegetables and low in salted, pickled, and smoked foods。
Eat small, frequent meals to avoid overloading the stomach。
Limit the intake of spicy, oily, and cold foods。
Ensure adequate protein intake from sources like fish, meat, eggs, and dairy。
Exercise management:
Engage in regular physical activity such as walking, jogging, or swimming to improve overall health。
Avoid excessive physical exertion and ensure adequate rest。
Maintain a regular sleep schedule to support the body's recovery。

10. What are the treatment methods for early gastric cancer?
Endoscopic resection: For small, localized tumors, endoscopic mucosal resection (EMR) or endoscopic submucosal dissection (ESD) can be used to remove the tumor.
Surgery: Partial gastrectomy (removal of part of the stomach) is often performed to remove early-stage tumors.
Follow-up: Regular endoscopic surveillance to monitor for recurrence.

11. What are the treatments methods for intermediate gastric cancer?
Surgery: Total or partial gastrectomy, often combined with lymph node dissection.
Chemotherapy: Before surgery (neoadjuvant) to shrink the tumor or after surgery (adjuvant) to kill any remaining cancer cells.
Radiation therapy: Sometimes used in combination with chemotherapy, especially for tumors that are difficult to remove completely.

12. What are the treatment methods for advanced gastric cancer?
Chemotherapy: To control the growth of the cancer and alleviate symptoms.
Targeted therapy: Drugs that target specific molecular changes in cancer cells, such as trastuzumab for HER2-positive gastric cancer.
Immunotherapy: Boosts the body's immune system to fight cancer cells.
Palliative care: Focuses on improving quality of life and managing symptoms such as pain, nausea, and difficulty eating.

13. What are the surgical methods of gastric cancer?
Partial gastrectomy: Removal of part of the stomach, often used for early-stage tumors.
Total gastrectomy: Removal of the entire stomach, used for more advanced or larger tumors.
Lymph node dissection: Removal of nearby lymph nodes to check for cancer spread and to reduce the risk of recurrence.
Reconstructive surgery: After gastrectomy, the surgeon may perform reconstructive surgery to help the digestive system function properly, such as an esophagojejunostomy.

14. What should I pay attention to after discharge from gastric cancer surgery?

Diet:
Start with a liquid diet and gradually transition to soft foods and then a regular diet.
Eat small, frequent meals to avoid overloading the stomach.
Avoid spicy, fatty, and hard-to-digest foods.
Activity:
Engage in light physical activity as tolerated to promote recovery.
Avoid heavy lifting or strenuous exercise for a period recommended by your doctor.
Follow-up:
Regular follow-up appointments are crucial to monitor recovery and detect any recurrence early.
Follow the recommended schedule for blood tests, imaging studies, and endoscopies.
Medication:
Continue any prescribed medications, including pain relievers and anti-nausea drugs.
Report any side effects to your healthcare provider.
Psychological support:
Seek emotional support from family, friends, or a support group.
Consider counseling if needed to cope with the emotional impact of the surgery and recovery .

15. What is the medication regimen for advanced gastric cancer?
Chemotherapy:
First-line treatment:
For HER2-negative tumors: Fluorouracil or capecitabine combined with oxaliplatin and nivolumab, or triplet regimens such as 5-FU with epirubicin and cisplatin .
For HER2-positive tumors: Trastuzumab, cisplatin, and capecitabine (HCX) .
Second-line treatment:
Ramucirumab with or without chemotherapy, pembrolizumab for patients with dMMR or MSI-high tumors, or trastuzumab deruxtecan for HER2-positive tumors .
Third-line treatment:
Regorafenib with nivolumab (under clinical evaluation) .
Targeted therapy:
Trastuzumab for HER2-positive gastric cancer .
Ramucirumab for anti-VEGF therapy .
Immunotherapy:
Nivolumab, pembrolizumab, and other immune checkpoint inhibitors are used to enhance the immune system's ability to attack cancer cells .

16. What is the principle of chemotherapy drug action in gastric cancer?
Fluorouracil (5-FU):
Inhibits thymidylate synthase, preventing DNA synthesis and repair, leading to cell death.
Oxaliplatin:
Forms DNA adducts, interfering with DNA replication and transcription, causing cell cycle arrest and apoptosis.
Docetaxel:
Promotes microtubule polymerization and prevents microtubule depolymerization, disrupting cell division and leading to cell death.

Epirubicin:
Intercalates into DNA, inhibiting topoisomerase II, leading to DNA damage and cell death.
Cisplatin:
Binds to DNA, forming intrastrand and interstrand crosslinks, preventing DNA replication and transcription, leading to cell death .

17. What are the side effects of commonly used chemotherapy drugs for advanced gastric cancer?
Fluorouracil (5-FU):
Nausea, vomiting, diarrhea, mouth sores, and hand-foot syndrome.
Oxaliplatin:
Nausea, vomiting, neuropathy (tingling or numbness in hands and feet), and allergic reactions.
Docetaxel:
Hair loss, nausea, vomiting, diarrhea, and increased risk of infection due to low white blood cell counts.
Epirubicin:
Nausea, vomiting, hair loss, and cardiac toxicity (monitoring is required).
Cisplatin:
Nausea, vomiting, kidney toxicity, and hearing loss.
Capecitabine:
Hand-foot syndrome, nausea, vomiting, and diarrhea

18. What are the options of immunotherapy for gastric cancer？
Immune checkpoint inhibitors:
PD-1 inhibitors: Nivolumab (Opdivo) and pembrolizumab (Keytruda) are used to target PD-1, a protein on T cells. By blocking PD-1, these drugs boost the immune response against cancer cells, which can shrink some tumors or slow their growth。
PD-L1 inhibitors: Atezolizumab and durvalumab are also used in some cases to target PD-L1, another checkpoint protein。
Combination therapies: Some studies are exploring the combination of immunotherapy with chemotherapy or targeted therapy to enhance the effectiveness of treatment。

19. What are the side effects of immunotherapy for gastric cancer？
Common side effects:Feeling tired or weak，Fever，Cough，Nausea，Itching，Skin rash
Loss of appetite，Muscle or joint pain，Shortness of breath，Constipation or diarrhea
Serious side effects:
Infusion reactions: These can include fever, chills, flushing of the face, rash, itchy skin, feeling dizzy, wheezing, and trouble breathing。
Autoimmune reactions: These drugs can sometimes cause the immune system to attack other parts of the body, leading to serious or even life-threatening problems in the lungs, intestines, liver, hormone-making glands, kidneys, skin, or other organs。

20. What are the targeted drugs for the treatment of gastric cancer？
Trastuzumab: Used for HER2-positive gastric cancer. It targets the HER2 protein, which is

overexpressed in some gastric cancers, and helps to block the growth of cancer cells。
Ramucirumab: Targets the VEGF receptor, inhibiting the formation of new blood vessels that supply the tumor with nutrients and oxygen。
Disitamab vedotin (RC48): An antibody-drug conjugate that selectively delivers cytotoxic agents to tumor sites, effectively killing tumor cells. It is approved for the treatment of HER2-positive advanced gastric cancer。

21. What are the side effects of targeted therapy for gastric cancer？
Trastuzumab:
Cardiac toxicity: Can affect heart function, requiring regular monitoring。
Infusion reactions: Similar to those seen with immunotherapy, including fever, chills, and rash。
Ramucirumab:
Hypertension: Can cause high blood pressure, which needs to be managed。
Proteinuria: Can lead to protein in the urine, indicating kidney issues。
Disitamab vedotin (RC48):
Peripheral neuropathy: Can cause numbness or tingling in the hands and feet。
Fatigue and nausea: Common side effects that can affect the patient's quality of life。

22. Whether radiotherapy is necessary for gastric cancer？
Radiotherapy is not always necessary for gastric cancer but can be an important part of the treatment plan in certain cases. It is often used in combination with chemotherapy (chemoradiotherapy) for locally advanced gastric cancer to shrink the tumor before surgery or to control the disease in patients who are not candidates for surgery. The decision to use radiotherapy depends on the stage of the cancer, the patient's overall health, and the specific treatment goals.

23. What are the side effects of radiotherapy for gastric cancer？
Radiotherapy for gastric cancer can cause several side effects, including:
Gastrointestinal symptoms: Nausea, vomiting, diarrhea, and loss of appetite.
Fatigue: A common side effect that can persist throughout the treatment period.
Skin changes: Redness, dryness, and irritation in the area where the radiation is directed.
Bone marrow suppression: Leading to a decrease in white blood cells, red blood cells, and platelets, which can increase the risk of infection, anemia, and bleeding.
Long-term effects: May include bowel obstruction, ulcers, or strictures.

24. How to evaluate the effectiveness of therapeutic regimen for gastric cancer？
To evaluate the effectiveness of a gastric cancer treatment plan simply:
Tumor Response: Check if the tumor shrinks or disappears. This is called Objective Response Rate (ORR).
Progression-Free Survival (PFS): Measure the time from treatment start to when the cancer gets worse.
Overall Survival (OS): Track the time from treatment start to when the patient passes away.
Quality of Life (QoL): See how the treatment affects the patient's daily life.

Side Effects (AEs): Look at any bad reactions from the treatment.
Biomarkers: Test for specific markers that can predict how well the treatment will work.
Real-World Data: Use studies to see how well the treatment works in real life.
Guidelines and Standards: Follow expert guidelines to make sure the treatment is good.

25. Can gastric cancer patients be cured, and is there a risk of recurrence after cure?
Cure possibility: Early-stage gastric cancer can often be cured with surgery, sometimes combined with chemotherapy or radiation therapy. The cure rate is higher for early-stage cancer.
Recurrence risk: Even after successful treatment, there is a risk of recurrence. Regular follow-up is crucial to detect any recurrence early and manage it effectively.

DeepSeek

1. What is gastric cancer?
Stomach cancer, also known as gastric cancer, is a type of cancer that develops in the lining of the stomach. It typically begins in the mucus-producing cells that line the stomach (adenocarcinoma), which is the most common form. Other rare types include lymphoma, gastrointestinal stromal tumors (GIST), and carcinoid tumors.

2. What causes gastric cancer?
The exact cause of stomach cancer is not fully understood, but several risk factors have been identified:
Helicobacter pylori infection: A bacterial infection that causes chronic inflammation and ulcers in the stomach lining.
Diet: High consumption of salted, smoked, or pickled foods and low intake of fruits and vegetables.
Tobacco and alcohol use: Both increase the risk of stomach cancer.
Family history: Genetic predisposition or inherited conditions like hereditary diffuse gastric cancer (HDGC).
Chronic gastritis: Long-term inflammation of the stomach lining.
Pernicious anemia: A condition that affects stomach lining cells.
Obesity: Increases the risk of cancers in the upper part of the stomach.
Previous stomach surgery: Such as surgery for ulcers, which may increase risk.
Age and gender: More common in older adults and men.

3. How is gastric cancer diagnosed?
Diagnosis involves a combination of tests and procedures:
Endoscopy: A thin, flexible tube with a camera (endoscope) is used to examine the stomach lining and take tissue samples (biopsy).
Biopsy: Tissue samples are analyzed under a microscope to confirm cancer.
Imaging tests: CT scans, PET scans, or X-rays to determine the extent of cancer spread.
Barium swallow: A patient drinks a liquid containing barium, which coats the stomach and makes it visible on X-rays.
Blood tests: To check for anemia or tumor markers.

Laparoscopy: A minimally invasive procedure to examine the abdomen and take tissue samples.

4. How can I prevent gastric cancer?
While not all cases are preventable, certain measures can reduce the risk:
Treat H. pylori infection: Antibiotics can eliminate the bacteria and reduce cancer risk.
Healthy diet: Eat more fruits, vegetables, and whole grains, and reduce intake of salted, smoked, or processed foods.
Avoid tobacco and limit alcohol: Both are significant risk factors.
Maintain a healthy weight: Obesity increases the risk of stomach cancer.
Regular screening: For individuals with a family history or genetic predisposition.

5. How to stage gastric cancer?
Staging determines the extent of cancer spread and guides treatment. The most common system is the TNM system:
T (Tumor): Size and extent of the primary tumor.
T1: Tumor invades the inner lining.
T2: Tumor invades the muscle layer.
T3: Tumor penetrates the outer layer.
T4: Tumor invades nearby organs.
N (Nodes): Whether cancer has spread to nearby lymph nodes.
N0: No lymph node involvement.

N1-N3: Increasing involvement of lymph nodes.
M (Metastasis): Whether cancer has spread to distant organs.
M0: No distant spread.
M1: Cancer has spread to distant organs.
Stages are grouped as:
Stage 0: Cancer is only in the inner lining (early stage).
Stage I: Cancer has spread to deeper layers but not to lymph nodes.
Stage II: Cancer has spread to deeper layers and nearby lymph nodes.
Stage III: Cancer has spread extensively to lymph nodes or nearby tissues.
Stage IV: Cancer has metastasized to distant organs (advanced stage).

6. What are the symptoms of gastric cancer?
Stomach cancer symptoms often do not appear in the early stages, but as the disease progresses, the following symptoms may occur:
Persistent indigestion or heartburn
Abdominal pain or discomfort, especially after eating
Nausea and vomiting, sometimes with blood
Difficulty swallowing (dysphagia)
Unexplained weight loss
Loss of appetite
Feeling full after eating small amounts (early satiety)
Fatigue or weakness due to anemia

Black stools (melena) or blood in the stool
Bloating or swelling in the abdomen
These symptoms can also be caused by other conditions, but if they persist, it is important to consult a doctor.

7. How long does gastric cancer patients survive?
Survival time for gastric cancer depends on the stage at diagnosis, overall health, and treatment effectiveness:
Early-stage (localized): If diagnosed at Stage I, the 5-year survival rate is around 70-90%.
Locally advanced: For Stage II and III, the 5-year survival rate drops to 30-50%.
Advanced-stage (metastatic): For Stage IV, the 5-year survival rate is less than 5-10%.
Early detection and treatment significantly improve survival rates.

8. Whether gastric cancer can be inherited, and whether relatives need testing?
Inherited risk: Most stomach cancers are not inherited, but about 1-3% of cases are linked to genetic mutations, such as:
CDH1 mutation: Causes hereditary diffuse gastric cancer (HDGC).
Lynch syndrome: Increases the risk of stomach and other cancers.
BRCA1/BRCA2 mutations: May slightly increase stomach cancer risk.
Family history: If multiple close relatives have had stomach cancer, genetic counseling and testing may be recommended.
Testing for relatives: If a genetic mutation is identified, first-degree relatives (parents, siblings, children) should consider genetic testing and regular screenings.

9. How to manage diet and exercise after having gastric cancer?
After a stomach cancer diagnosis, maintaining proper nutrition and physical activity is crucial for recovery and overall well-being:
Dietary management:
Small, frequent meals: Eat 5-6 small meals daily to avoid overloading the stomach.
High-protein foods: Include lean meats, fish, eggs, beans, and dairy to support healing.
Soft, easy-to-digest foods: Opt for cooked vegetables, soups, and pureed foods if swallowing is difficult.
Avoid irritants: Limit spicy, acidic, or fried foods that may cause discomfort.
Stay hydrated: Drink water and clear fluids throughout the day.
Vitamin supplements: If nutrient absorption is impaired (e.g., after surgery), take prescribed supplements like vitamin B12, iron, or calcium.
Exercise:
Light to moderate activity: Walking, yoga, or stretching can improve energy levels and reduce fatigue.
Avoid overexertion: Listen to your body and rest when needed.
Physical therapy: If surgery or treatment has affected mobility, consult a physical therapist for a tailored plan.
Work with a dietitian: A registered dietitian can help create a personalized nutrition plan based on your treatment and recovery needs.

10. What are the treatment methods for early gastric cancer?
Early gastric cancer (Stage 0 or Stage I) is highly treatable, and options include:
Endoscopic resection:
Endoscopic mucosal resection (EMR): Removes small, early-stage tumors confined to the stomach lining.
Endoscopic submucosal dissection (ESD): Used for slightly larger tumors that haven’t spread deeper.
Surgery:
Partial gastrectomy: Removal of part of the stomach containing the tumor.
Lymph node dissection: Nearby lymph nodes may be removed to check for cancer spread.
Adjuvant therapy:
Chemotherapy or radiation may be recommended after surgery to eliminate remaining cancer cells.

11. What are the the treatments methods for intermediate gastric cancer?
Mid-stage gastric cancer (Stage II or III) typically requires a combination of treatments:
Surgery:
Total or partial gastrectomy: Removal of part or all of the stomach, depending on the tumor's location and size.
Lymph node dissection: Removal of nearby lymph nodes to prevent spread.
Neoadjuvant therapy:
Chemotherapy or chemoradiation before surgery to shrink the tumor.
Adjuvant therapy:
Chemotherapy or radiation after surgery to kill remaining cancer cells.
Targeted therapy:
For HER2-positive tumors, drugs like trastuzumab may be used.

12. What are the treatment methods for advanced gastric cancer?
Advanced gastric cancer (Stage IV) involves cancer that has spread to distant organs. Treatment focuses on controlling symptoms and prolonging life:
Chemotherapy:
The primary treatment for advanced cancer, often using combinations like FLOT (5-FU, leucovorin, oxaliplatin, docetaxel).
Targeted therapy:
HER2-targeted drugs: Trastuzumab for HER2-positive cancers.
VEGF inhibitors: Ramucirumab to block blood vessel growth in tumors.
Immunotherapy:
PD-1 inhibitors: Pembrolizumab or nivolumab for cancers with specific biomarkers (e.g., MSI-H or PD-L1 expression).
Palliative care:
Surgery, radiation, or stents to relieve symptoms like blockages or bleeding.
Pain management and nutritional support to improve quality of life.

13. What are the surgical methods of gastric cancer?
Surgery is a key treatment for stomach cancer, and the type depends on the tumor's location and stage:
Endoscopic resection:
Used for very early-stage cancers (Stage 0 or early Stage I).
Includes EMR and ESD.
Partial gastrectomy:
Removal of the lower part of the stomach (distal gastrectomy) or upper part (proximal gastrectomy).
Often used for tumors in specific areas of the stomach.
Total gastrectomy:
Removal of the entire stomach, often for large or centrally located tumors.
The esophagus is connected directly to the small intestine.
Lymph node dissection:
Removal of nearby lymph nodes to check for cancer spread (D1 or D2 dissection).
Palliative surgery:
For advanced cancer, surgery may be used to relieve symptoms (e.g., removing blockages or placing stents).

14. What should I pay attention to after discharge from gastric cancer surgery?
Diet:
Start with small, frequent meals.
Avoid spicy, fatty, or hard-to-digest foods.
Gradually reintroduce solid foods as tolerated.
Monitor for dumping syndrome (rapid gastric emptying), which can cause nausea, diarrhea, and dizziness.
Wound Care:
Keep the surgical site clean and dry.
Watch for signs of infection (redness, swelling, discharge).
Activity:
Avoid heavy lifting or strenuous activities for several weeks.
Gradually increase physical activity as tolerated.
Medications:
Take prescribed medications (e.g., PPIs, pain relievers) as directed.
Do not skip adjuvant chemotherapy or follow-up treatments.
Follow-Up Appointments:
Regular check-ups to monitor recovery and detect recurrence.
Imaging tests (CT scans) and endoscopies as recommended.
Symptoms to Report:
Persistent pain, vomiting, difficulty swallowing, or unexplained weight loss.
Emotional Support:
Seek counseling or support groups to cope with emotional and psychological challenges.

15. What is the medication regimen for advanced gastric cancer?

Chemotherapy:
Combination therapies like FLOT (5-FU, leucovorin, oxaliplatin, docetaxel) or FOLFOX (5-FU, leucovorin, oxaliplatin).
Single-agent chemotherapy (e.g., capecitabine) for frail patients.
Targeted Therapy:
Trastuzumab for HER2-positive tumors.
Ramucirumab (anti-VEGFR2) used alone or with paclitaxel.
Pembrolizumab (immunotherapy) for tumors with high microsatellite instability (MSI-H) or PD-L1 expression.
Immunotherapy:
Nivolumab or pembrolizumab for advanced or metastatic gastric cancer.
Palliative Care:
Pain management with opioids or non-opioid analgesics.
Antiemetics for nausea and vomiting.
Nutritional support and supplements.

16. What is the principle of chemotherapy drug action in gastric cancer?
Chemotherapy drugs work by targeting rapidly dividing cells, including cancer cells. Their mechanisms include:
Inhibition of DNA Synthesis: Drugs like 5-FU and capecitabine interfere with DNA replication.
DNA Damage: Platinum-based drugs (e.g., cisplatin, oxaliplatin) cause DNA cross-linking, preventing cell division.
Microtubule Inhibition: Taxanes (e.g., docetaxel, paclitaxel) disrupt the cell's microtubule structure, preventing mitosis.
Topoisomerase Inhibition: Drugs like irinotecan inhibit topoisomerase enzymes, causing DNA breaks.
Antimetabolites: Drugs like 5-FU mimic natural metabolites, disrupting RNA and DNA synthesis.

17. What are the side effects of commonly used chemotherapy drugs for advanced gastric cancer?
5-Fluorouracil (5-FU):
Nausea, vomiting, diarrhea.
Mouth sores.
Bone marrow suppression (anemia, leukopenia, thrombocytopenia).
Hand-foot syndrome (redness, swelling, pain in palms and soles).
Capecitabine:
Similar to 5-FU, including diarrhea, hand-foot syndrome, and bone marrow suppression.
Fatigue and loss of appetite.
Cisplatin:
Kidney damage (nephrotoxicity).
Hearing loss (ototoxicity).
Peripheral neuropathy (numbness, tingling in hands and feet).
Severe nausea and vomiting.
Oxaliplatin:

Peripheral neuropathy (often triggered by cold).
Nausea, vomiting, and diarrhea.
Bone marrow suppression.
Docetaxel:
Bone marrow suppression.
Fluid retention and swelling.
Hair loss.
Fatigue and muscle pain.
Paclitaxel:
Bone marrow suppression.
Peripheral neuropathy.
Hair loss.
Allergic reactions.
Trastuzumab:
Cardiotoxicity (heart failure risk).
Infusion-related reactions (fever, chills).
Fatigue and diarrhea.
Ramucirumab:
Hypertension.
Fatigue.
Bleeding and wound healing complications.
Proteinuria (protein in urine).
Pembrolizumab/Nivolumab:
Immune-related adverse events (e.g., colitis, hepatitis, pneumonitis).
Fatigue, rash, and itching.
Endocrine disorders (e.g., thyroid dysfunction).

18. What are the options of immunotherapy for gastric cancer?
Immunotherapy is a treatment option for gastric cancer, particularly for advanced or metastatic cases. The main types of immunotherapy used include:
Immune Checkpoint Inhibitors:
Pembrolizumab (Keytruda): Approved for gastric cancer with high microsatellite instability (MSI-H) or PD-L1 expression.
Nivolumab (Opdivo): Used in combination with chemotherapy or as monotherapy for advanced gastric cancer.
Ipilimumab (Yervoy): Sometimes used in combination with other immunotherapies.
CAR-T Cell Therapy:
Still under investigation for gastric cancer, but shows promise in targeting specific tumor antigens.
Cancer Vaccines:
Experimental vaccines targeting specific gastric cancer antigens are being studied in clinical trials.

19. What are the side effects of immunotherapy for gastric cancer?
Immunotherapy can cause immune-related adverse events (irAEs) due to the activation of the immune system against normal tissues. Common side effects include:

Fatigue: Mild to severe tiredness.
Skin Reactions: Rash, itching, or vitiligo (loss of skin pigmentation).
Gastrointestinal Issues: Diarrhea, colitis, or abdominal pain.
Endocrine Disorders: Hypothyroidism, hyperthyroidism, or adrenal insufficiency.
Liver Toxicity: Hepatitis, leading to elevated liver enzymes.
Pneumonitis: Inflammation of the lungs, causing cough or shortness of breath.
Kidney Issues: Nephritis, leading to reduced kidney function.
Infusion Reactions: Fever, chills, or allergic reactions during or after infusion.
Muscle or Joint Pain: Myalgia or arthralgia.
Severe side effects require prompt medical attention and may involve temporary or permanent discontinuation of immunotherapy.

20. What are the targeted drugs for gastric cancer treatment?
Targeted therapy focuses on specific molecular targets involved in cancer growth and progression. The main targeted drugs for gastric cancer include:
HER2-Targeted Therapy:
Trastuzumab (Herceptin): For HER2-positive gastric cancer.
Fam-trastuzumab deruxtecan (Enhertu): An antibody-drug conjugate for HER2-positive advanced gastric cancer.
Anti-Angiogenic Therapy:
Ramucirumab (Cyramza): Targets VEGFR2 to inhibit blood vessel formation in tumors. Used alone or with paclitaxel.
EGFR Inhibitors:
Cetuximab (Erbitux): Under investigation for gastric cancer, but not widely used.
mTOR Inhibitors:
Everolimus (Afinitor): Used in some cases of advanced gastric cancer.
PARP Inhibitors:
Olaparib (Lynparza): Investigated for gastric cancer with DNA repair deficiencies.
Claudin 18.2-Targeted Therapy:
Zolbetuximab: A monoclonal antibody targeting Claudin 18.2, currently in clinical trials.

21. What are the side effects of targeted therapy for gastric cancer?
Targeted therapies have specific side effects based on their mechanism of action:
Trastuzumab:
Cardiotoxicity (heart failure risk).
Infusion-related reactions (fever, chills).
Fatigue, diarrhea, and nausea.
Ramucirumab:
Hypertension.
Fatigue.
Bleeding and wound healing complications.
Proteinuria (protein in urine).
Fam-trastuzumab deruxtecan:
Nausea, vomiting, and fatigue.

Bone marrow suppression (low blood cell counts).
Interstitial lung disease (a serious lung condition).
Everolimus:
Mouth sores.
Fatigue and rash.
Hyperglycemia (high blood sugar).
Increased risk of infections.
Cetuximab:
Skin rash (acne-like).
Hypomagnesemia (low magnesium levels).
Infusion reactions.
Olaparib:
Fatigue and nausea.
Bone marrow suppression.
Increased risk of blood clots

22. Whether radiotherapy is necessary for gastric cancer?
Radiotherapy may or may not be necessary for gastric cancer, depending on the stage, location, and treatment plan.
Early-stage gastric cancer: Radiotherapy is not typically used as a primary treatment but may be combined with chemotherapy (chemoradiation) before or after surgery to reduce the risk of recurrence.
Locally advanced gastric cancer: Radiotherapy is often used in combination with chemotherapy to shrink tumors before surgery or to target remaining cancer cells after surgery.
Advanced or metastatic gastric cancer: Radiotherapy is primarily used for palliative purposes to relieve symptoms such as pain, bleeding, or obstruction.
The decision to use radiotherapy is made by the oncologist based on the individual patient’s condition and treatment goals.

23. What are the side effects of radiotherapy for gastric cancer?
Radiotherapy for gastric cancer can cause both short-term and long-term side effects, which vary depending on the dose, duration, and area treated. Common side effects include:
Short-term side effects:
Fatigue
Nausea and vomiting
Loss of appetite
Diarrhea or abdominal discomfort
Skin irritation or redness in the treated area
Difficulty swallowing (if the esophagus is affected)
Long-term side effects:
Scarring or fibrosis of surrounding tissues
Damage to nearby organs (e.g., liver, kidneys, or intestines)
Increased risk of secondary cancers in the irradiated area
Chronic digestive issues, such as malabsorption or bowel obstruction

Patients should discuss potential side effects with their healthcare team and report any symptoms promptly for management.

24. How to evaluate the effectiveness of therapeutic regimens for gastric cancer?
The effectiveness of therapeutic regimens for gastric cancer can be evaluated using several key metrics:
Patient outcomes: Survival rates, recurrence rates, and quality of life after treatment.
Treatment adherence: Compliance with evidence-based guidelines for surgery, chemotherapy, radiotherapy, and follow-up care.
Timeliness of care: Speed of diagnosis, initiation of treatment, and access to specialized care.
Patient satisfaction: Feedback from patients regarding their experience with care, communication, and support.
Complication rates: Frequency of post-treatment complications, such as infections or readmissions.
Cost-effectiveness: Efficiency of resource use and affordability of care for patients.
Regular audits, data collection, and benchmarking against national or international standards are essential for continuous improvement.

25. Can gastric cancer patients be cured, and is there a risk of recurrence after cure?
Curability:
Early-stage gastric cancer (localized to the stomach) can often be cured with surgery, sometimes combined with chemotherapy or radiotherapy.
Advanced-stage gastric cancer is less likely to be cured, but treatment can control the disease, relieve symptoms, and prolong survival.
Risk of recurrence:
Even after successful treatment, there is a risk of recurrence, especially in the first 2 – 3 years.
The risk depends on factors such as the stage at diagnosis, the completeness of surgical removal, and the response to adjuvant therapy.
Regular follow-up is crucial to detect and manage recurrence early.

ChatGPT

1. What is gastric cancer?
Stomach cancer, also known as gastric cancer, is cancer that begins in the lining of the stomach. It can grow and spread to other organs. Most stomach cancers are adenocarcinomas, which start in the cells that produce mucus and other fluids in the stomach.

2. What causes gastric cancer?
The exact cause of stomach cancer is not fully understood, but several factors increase the risk:
Helicobacter pylori infection: A bacteria that causes stomach inflammation.
Diet: Eating too much salty, smoked, or pickled food.
Smoking and excessive alcohol use.

Family history: A family history of stomach cancer may increase the risk.
Age and gender: Older adults, especially men, are at higher risk.

3. How is gastric cancer diagnosed?
Stomach cancer is diagnosed through:
Endoscopy: A tube with a camera is inserted through the mouth to examine the stomach.
Biopsy: A tissue sample is taken from the stomach lining during endoscopy to check for cancer cells.
Imaging tests: CT scans, MRIs, or X-rays help determine the size and spread of the cancer.

4. How can I prevent gastric cancer?
While not all cases can be prevented, you can reduce your risk by:
Eating a healthy diet: Focus on fruits, vegetables, and whole grains.
Avoiding smoking and limiting alcohol consumption.
Treating H. pylori infections with antibiotics.
Maintaining a healthy weight and regular exercise.

5. How to stage gastric cancer?
Staging describes how far the cancer has spread. It is done through:
Endoscopy and biopsy: To check the size and location of the tumor.
Imaging tests: CT scans and ultrasounds to see if the cancer has spread to other organs.
The stages range from Stage 1 (early, localized) to Stage 4 (advanced, spread to other parts of the body).

6. What are the symptoms of gastric cancer?
Stomach cancer often has vague and nonspecific symptoms, especially in the early stages, which can make it difficult to diagnose. Common symptoms include:
Abdominal pain: Persistent discomfort, bloating, or a feeling of fullness.
Indigestion or heartburn: Persistent acid reflux or difficulty swallowing.
Loss of appetite: Decreased desire to eat or feeling full after eating small amounts.
Weight loss: Unexplained weight loss, often due to loss of appetite or difficulty eating.
Nausea and vomiting: Especially if the tumor obstructs the stomach.
Fatigue: Feeling unusually tired or weak.
Blood in stool or vomit: Can appear as dark, tarry stools or vomiting blood (indicating bleeding in the stomach).
Anemia: Low red blood cell count due to internal bleeding.
These symptoms can also be caused by conditions other than stomach cancer, so it's important to consult a healthcare provider for proper diagnosis.

7. How long does gastric cancer patients survive?
The survival time for gastric cancer depends on several factors, including the stage of cancer at diagnosis, the patient's overall health, and response to treatment.
Early-stage gastric cancer: If caught early and treated effectively (often by surgery), the 5-year survival rate can be as high as 90%.

Advanced-stage gastric cancer: The prognosis is less favorable, with the 5-year survival rate dropping to about 20% or lower for metastatic cases.
However, survival rates can vary widely. Many people with advanced gastric cancer may live for years with appropriate treatment, including chemotherapy, immunotherapy, and palliative care.

8. Whether gastric cancer can be inherited, and whether relatives need testing?
Inheritance:
Gastric cancer can have a hereditary component, especially in families with hereditary diffuse gastric cancer (HDGC) or conditions like Lynch syndrome. People with these genetic conditions are at higher risk for stomach cancer.
In most cases, however, gastric cancer is sporadic and not inherited.
Family testing:
Relatives of people with a known hereditary risk may benefit from genetic counseling and testing to assess their risk. Early screening may be recommended for high-risk individuals, such as those with a strong family history of stomach cancer or known inherited syndromes. Regular screenings, like endoscopy, may help detect early signs of cancer in at-risk individuals.

9. How to manage diet and exercise after having gastric cancer?
After a stomach cancer diagnosis and treatment, it's important to:
Follow a balanced diet: Focus on eating small, frequent meals with healthy foods like fruits, vegetables, lean proteins, and whole grains.
Avoid heavy or spicy foods: These can irritate the stomach.
Stay hydrated and consider working with a nutritionist for meal planning.
Exercise: Moderate activity, like walking, can help improve energy levels and overall health. Always check with your doctor before starting an exercise plan.

10. What are the treatment methods for early gastric cancer?
Treatment for early gastric cancer includes:
Endoscopic submucosal dissection (ESD): A minimally invasive procedure for tumors confined to the mucosal layer.
Surgical resection: Partial gastrectomy, removing the cancerous part of the stomach.
Chemotherapy or immunotherapy: Sometimes used as an adjuvant treatment, depending on the tumor's characteristics.

11. What are the treatments methods for intermediate gastric cancer?
Treatment for mid-stage gastric cancer typically involves:
Surgical resection: Gastrectomy (partial or total) with lymph node dissection to remove the tumor and affected lymph nodes.
Chemotherapy: Often used before (neoadjuvant) or after surgery (adjuvant) to shrink the tumor and reduce the risk of recurrence.
Radiation therapy: May be combined with chemotherapy for some patients to improve outcomes.

12. What are the treatment methods for advanced gastric cancer?

Treatment for advanced gastric cancer includes:
Chemotherapy: The mainstay treatment for advanced gastric cancer to control the disease and alleviate symptoms.
Targeted therapy: Drugs that target specific molecules involved in cancer cell growth (e.g., HER2-targeted therapy for HER2-positive tumors).
Immunotherapy: PD-1 inhibitors and other immune checkpoint inhibitors may be used for certain patients.
Palliative care: To manage symptoms and improve quality of life when the cancer cannot be cured.

13. What are the surgical methods of gastric cancer?
Surgical methods for gastric cancer depend on the stage and location of the tumor:
Gastrectomy: Removal of part or all of the stomach. This is the most common surgery for gastric cancer.
Partial gastrectomy: Removal of the tumor and a portion of the stomach.
Total gastrectomy: Removal of the entire stomach, often with reconstruction of the digestive tract.
Lymph node dissection: Removal of nearby lymph nodes to check for spread of cancer.
Minimally invasive surgery (laparoscopy): In some cases, a minimally invasive technique may be used to remove the tumor with smaller incisions.

14. What should I pay attention to after discharge from gastric cancer surgery?
Diet: Start with liquids, then soft foods, gradually progressing to solid foods as tolerated. Small, frequent meals are recommended to avoid overloading the stomach.
Signs of Infection: Watch for fever, redness, or swelling at the surgical site.
Hydration: Ensure adequate fluid intake to prevent dehydration.
Activity Level: Avoid heavy lifting and strenuous activities until cleared by the doctor.
Follow-up Appointments: Attend scheduled follow-ups to monitor healing and detect any recurrence of cancer.
Weight Loss & Nutritional Support: Malnutrition can occur, so be mindful of weight loss and discuss nutritional supplements or specialized diets with your healthcare provider.

15. What is the medication regimen for advanced gastric cancer?
For advanced gastric cancer, treatment typically involves a combination of chemotherapy, targeted therapy, and sometimes immunotherapy.
Chemotherapy drugs: Such as fluorouracil (5-FU), cisplatin, oxaliplatin, or capecitabine.
Targeted therapy: Trastuzumab (Herceptin) for HER2-positive cancers, ramucirumab for advanced stages.
Immunotherapy: Checkpoint inhibitors like pembrolizumab (Keytruda) or nivolumab (Opdivo) may be used in some cases.

16. What is the principle of chemotherapy drug action in gastric cancer?
Chemotherapy drugs for gastric cancer work by targeting and killing fast-growing cancer cells. These drugs interfere with DNA replication or cellular division, inhibiting the growth and spread

of cancer cells. They may also enhance the immune system's ability to recognize and attack cancer cells. Commonly used drugs like 5-FU, cisplatin, and oxaliplatin disrupt cancer cell processes, leading to their death or halting their growth.

17. What are the side effects of commonly used chemotherapy drugs for advanced gastric cancer?
Fluorouracil (5-FU): Nausea, vomiting, mouth sores, diarrhea, fatigue, low blood counts (which may lead to increased risk of infection or bleeding).
Cisplatin: Kidney toxicity, hearing loss, nausea, vomiting, fatigue, and nerve damage.
Oxaliplatin: Neuropathy (nerve damage), nausea, vomiting, diarrhea, fatigue, and low blood counts.
Capecitabine: Nausea, vomiting, hand-foot syndrome (redness, swelling, and pain on palms and soles), fatigue, and low blood counts.
Trastuzumab (Herceptin): Heart problems (such as reduced heart function), fever, chills, nausea, and fatigue.
Ramucirumab: High blood pressure, fatigue, diarrhea, and risk of bleeding.
These side effects vary between individuals, and supportive care is often given to manage them.

18. What are the options of immunotherapy for gastric cancer?
Immunotherapy for gastric cancer includes:
Immune checkpoint inhibitors: These drugs help the immune system recognize and attack cancer cells. Common immune checkpoint inhibitors used for gastric cancer are:
Pembrolizumab (Keytruda): A PD-1 inhibitor that helps the immune system recognize cancer cells.
Nivolumab (Opdivo): Another PD-1 inhibitor used in advanced gastric cancer.
Atezolizumab (Tecentriq): A PD-L1 inhibitor, sometimes used for gastric cancer.
Monoclonal antibodies: Targeting specific molecules to activate the immune system, such as trastuzumab (Herceptin) for HER2-positive gastric cancer.
Cytokine therapy: In some cases, interleukin-2 (IL-2) or interferons may be used to stimulate immune activity against the tumor.
Chimeric Antigen Receptor (CAR) T-cell therapy: A newer approach involving engineering T-cells to attack cancer cells, though it is still experimental for gastric cancer.

19. What are the side effects of immunotherapy for gastric cancer?
The side effects of immunotherapy for gastric cancer can vary but typically include:
Fatigue: A common side effect of many immunotherapies.
Skin reactions: Rash, itching, or redness.
Gastrointestinal issues: Diarrhea, nausea, or colitis (inflammation of the colon).
Endocrine issues: Inflammation of the thyroid, pituitary, or adrenal glands, potentially leading to hormonal imbalances.
Pneumonitis: Inflammation of the lungs, causing difficulty breathing and coughing.
Hepatitis: Liver inflammation, which can lead to jaundice or liver damage.

Autoimmune reactions: Since immunotherapy boosts the immune system, it can lead to the immune system attacking healthy cells, causing autoimmune diseases.
Severe side effects are rare but can be serious, including organ inflammation or severe reactions to certain immune checkpoint inhibitors.

20. What are the targeted drugs for the treatment of gastric cancer?
Targeted therapy for gastric cancer involves drugs that specifically target the molecules involved in cancer cell growth and survival, such as:
Trastuzumab (Herceptin): Targets HER2 (human epidermal growth factor receptor 2), overexpressed in some gastric cancers.
Ramucirumab (Cyramza): Targets VEGFR-2 (vascular endothelial growth factor receptor), inhibiting the formation of blood vessels that supply the tumor.
Lapatinib: An inhibitor of HER2, used in combination with other treatments for HER2-positive gastric cancer.
Pertuzumab (Perjeta): Used in combination with trastuzumab for HER2-positive gastric cancer.
Regorafenib (Stivarga): An oral multikinase inhibitor that targets various receptors involved in cancer growth.
Apatinib: A VEGFR-2 inhibitor used in certain cases of advanced gastric cancer.

21. What are the side effects of targeted therapy for gastric cancer?
Targeted therapy for gastric cancer has its own set of potential side effects, which may include:
Fatigue: One of the most common side effects.
Hypertension: High blood pressure, especially with drugs like ramucirumab or apatinib.
Diarrhea: Particularly with drugs like regorafenib.
Skin issues: Rash, hand-foot syndrome (redness and pain in the palms of hands and soles of feet).
Liver toxicity: Elevated liver enzymes or liver damage.
Mouth sores: Inflammation or sores in the mouth.
Gastrointestinal issues: Nausea, vomiting, or abdominal pain.
Bleeding: In some cases, targeted therapies may increase the risk of bleeding or clotting problems.
Both immunotherapy and targeted therapy can have serious side effects, so it's important for patients to be closely monitored during treatment.

22. Whether radiotherapy is necessary for gastric cancer?Radiotherapy is not always necessary but may be used in certain situations:
After surgery to eliminate remaining cancer cells
To relieve symptoms in advanced cancer
To shrink tumors before surgery

23. What are the side effects of radiotherapy for gastric cancer?
Side effects of radiotherapy for gastric cancer may include:
Fatigue
Nausea and vomiting
Skin irritation

Loss of appetite
Diarrhea or constipation
Difficulty swallowing
Long-term risk of secondary cancers

24. How to evaluate the effectiveness of therapeutic regimens for gastric cancer?
Effectiveness can be assessed by:
Survival and recurrence rates
Treatment success (tumor reduction or removal)
Patient quality of life
Adherence to clinical guidelines
Patient satisfaction
Treatment accessibility and cost-effectiveness

25. Can gastric cancer patients be cured, and is there a risk of recurrence after cure?
Early-stage gastric cancer can often be cured, especially with surgery.
However, there is still a risk of recurrence, particularly in advanced cases. Regular follow-up is important.

Gemini

1. What is gastric cancer?
Stomach cancer, also known as gastric cancer, is the development of malignant (cancerous) cells in the lining of the stomach. The stomach is a vital organ in the digestive system, responsible for breaking down food. Stomach cancer typically develops slowly and may not cause noticeable symptoms in the early stages.

2. What causes gastric cancer?
Helicobacter pylori (H. pylori) infection: This bacterium is a major risk factor for stomach cancer.
Chronic gastritis: Long-term inflammation of the stomach lining can increase the risk.
Pernicious anemia: A condition where the body cannot produce enough healthy red blood cells.
Family history: Having a close relative with stomach cancer increases the risk.
Smoking: Smoking significantly increases the risk of stomach cancer.
Diet: A diet high in smoked, salted, or pickled foods may increase the risk.
Obesity: Being overweight or obese may slightly increase the risk.
Environmental factors: Exposure to certain chemicals or pollutants may increase the risk.

3. How is gastric cancer diagnosed?
Endoscopy: A thin, flexible tube with a light and camera is inserted into the mouth and down the esophagus to examine the stomach lining. Biopsies (tissue samples) may be taken during this

procedure.
Upper endoscopy with biopsy: This is the most common diagnostic method.
Imaging tests:
X-ray: Can detect abnormalities in the stomach.
CT scan: Creates detailed images of the abdomen to assess the extent of the cancer.
PET scan: Uses radioactive glucose to detect areas of increased metabolic activity, which can help identify cancer.
Blood tests: May be used to check for anemia, inflammation, or the presence of certain tumor markers.

4. How can I prevent gastric cancer?
H. pylori eradication: Treating H. pylori infection can significantly reduce the risk of stomach cancer.
Healthy diet: Eat a balanced diet rich in fruits, vegetables, and whole grains. Limit consumption of processed foods, smoked foods, and red meat.
Maintain a healthy weight: Maintain a healthy weight through a balanced diet and regular exercise.
Quit smoking: Smoking significantly increases the risk of stomach cancer.
Limit alcohol consumption: Excessive alcohol consumption can increase the risk.

5. How to stage gastric cancer?
The stage of stomach cancer describes how far the cancer has spread. Staging helps determine the best treatment plan and predict the prognosis (outlook). The most common staging system is the TNM system:

T (Tumor): Describes the size and location of the primary tumor.
N (Nodes): Indicates whether the cancer has spread to nearby lymph nodes.
M (Metastasis): Indicates whether the cancer has spread to distant organs (e.g., liver, lungs).
The stages range from 0 (early stage) to IV (advanced stage). Higher stages generally indicate more aggressive cancer and a poorer prognosis.

6.What are the symptoms of gastric cancer?
Early stages: Often no symptoms.
Later stages: Digestive issues: Indigestion, heartburn, feeling full quickly, bloating, nausea, vomiting. Pain: Abdominal pain or discomfort. Weight loss: Unexplained weight loss. Anemia: Fatigue, weakness due to iron deficiency. Difficulty swallowing: (Dysphagia) .Vomiting blood: Or black, tarry stools (melena) due to internal bleeding.

7.How long does gastric cancer patients survive?
Early stages: Often no symptoms.
Later stages: Digestive issues: Indigestion, heartburn, feeling full quickly, bloating, nausea, vomiting. Pain: Abdominal pain or discomfort. Weight loss: Unexplained weight loss. Anemia: Fatigue, weakness due to iron deficiency. Difficulty swallowing: (Dysphagia) .Vomiting blood: Or black, tarry stools (melena) due to internal bleeding.

8.Whether gastric cancer can be inherited, and whether relatives need testing?
Genetics play a role: Some types of stomach cancer have a genetic component.
Family history increases risk: If you have a close relative with stomach cancer, your risk is higher.
Genetic testing: May be recommended in some cases, especially if you have a strong family history.

9.How to manage diet and exercise after having gastric cancer?
Diet: Small, frequent meals: May be easier to tolerate.
Bland foods: Avoid spicy, greasy, or acidic foods.
High-protein foods: To maintain strength.
Hydration: Drink plenty of fluids.
Exercise: Consult your doctor: For personalized recommendations.
Start slowly: Begin with gentle activities like walking.
Listen to your body: Rest when needed.

10.What are the treatment methods for early gastric cancer?
Endoscopic mucosal resection (EMR): For very early cancers confined to the inner lining of the stomach.
Endoscopic submucosal dissection (ESD): For slightly deeper cancers.
Partial gastrectomy: Surgical removal of part of the stomach.

11.What are the treatments methods for intermediate gastric cancer?
Surgery: Typically involves removing the affected portion of the stomach, nearby lymph nodes, and sometimes other organs.
Chemotherapy: May be given before (neoadjuvant) or after (adjuvant) surgery to improve outcomes.

12.What are the treatment methods for advanced gastric cancer?
Chemotherapy: Often used as the primary treatment or in combination with other therapies.
Immunotherapy: Medications that boost the body's immune system to fight cancer.
Targeted therapy: Drugs that target specific weaknesses in cancer cells.
Radiation therapy: May be used in some cases to shrink tumors or relieve symptoms.
Palliative care: Focuses on managing symptoms and improving quality of life when a cure is not possible.

13.What are the surgical methods of gastric cancer?
Total gastrectomy: Removal of the entire stomach.
Partial gastrectomy: Removal of part of the stomach, including:
Distal gastrectomy: Removal of the lower part of the stomach.
Subtotal gastrectomy: Removal of a larger portion of the stomach.
Lymph node dissection: Removal of lymph nodes near the stomach to check for cancer spread.
Reconstructive surgery: To reconnect the remaining parts of the digestive system after surgery.

14.What should I pay attention to after discharge from gastric cancer surgery?
Wound care: Keep the incision clean and dry.Diet: Follow your doctor's dietary recommendations. Start with a bland diet and gradually introduce new foods.
Medications: Take medications as prescribed by your doctor.
Rest: Get plenty of rest and avoid strenuous activity.
Follow-up appointments: Keep all scheduled follow-up appointments with your doctor.
Monitor for complications: Watch for signs of infection, bleeding, or other complications.

15.What is the medication regimen for advanced gastric cancer?
Chemotherapy: Often the first-line treatment, using various combinations of drugs.
Immunotherapy: Medications that boost the body's immune system to fight cancer.
Targeted therapy: Drugs that target specific weaknesses in cancer cells.
Combination therapy: May involve a combination of chemotherapy, immunotherapy, and/or targeted therapy.

16. What is the principle of chemotherapy drug action in gastric cancer?
Interfere with cell growth and division: Chemotherapy drugs work by interfering with the processes that cancer cells use to grow and divide.
Damage DNA: Some drugs damage the DNA of cancer cells, preventing them from replicating.
Interfere with protein synthesis: Some drugs interfere with the production of proteins that cancer cells need to survive.

17.What are the side effects of commonly used chemotherapy drugs for advanced gastric cancer？
Nausea and vomiting, Fatigue, Hair loss. Mouth sores. Diarrhea or constipation, Bone marrow suppression (leading to low blood cell counts), Neurological problems (numbness, tingling)

18.What are the options of immunotherapy for gastric cancer？
Checkpoint inhibitors: These drugs block proteins on cancer cells and immune cells, allowing the immune system to better recognize and attack the cancer.
PD-1 inhibitors: Such as nivolumab (Opdivo) and pembrolizumab (Keytruda).
PD-L1 inhibitors: Such as atezolizumab (Tecentriq).

19.What are the side effects of immunotherapy for gastric cancer？
Immune-related adverse events (irAEs): These occur when the immune system attacks healthy tissues in the body.
Pneumonitis: Inflammation of the lungs.
Colitis: Inflammation of the colon.
Hepatitis: Inflammation of the liver.
Endocrinopathies: Problems with hormone production.
Skin rashes
Fatigue

20.What are the targeted drugs for the treatment of gastric cancer？
HER2-targeted therapy: For gastric cancers that overexpress the HER2 protein.
Trastuzumab (Herceptin)
Trastuzumab emtansine (Kadcyla)
VEGF-targeted therapy: Bevacizumab (Avastin) targets vascular endothelial growth factor, which helps prevent the growth of new blood vessels that tumors need to survive.
EGFR-targeted therapy: For gastric cancers with specific genetic mutations.

21.What are the side effects of targeted therapy for gastric cancer？
HER2-targeted therapy: Heart problems: Can weaken the heart muscle.
VEGF-targeted therapy: High blood pressure, Bleeding problems, Slow wound healing
EGFR-targeted therapy: Skin rash, Diarrhea, Fatigue

22.Whether radiotherapy is necessary for gastric cancer？
Radiotherapy may be used in certain situations:
Neoadjuvant radiotherapy: Given before surgery to shrink the tumor and improve surgical outcomes.
Adjuvant radiotherapy: Given after surgery to destroy any remaining cancer cells.
Palliative radiotherapy: Used to relieve symptoms such as pain or bleeding in advanced-stage cancer.
Decision based on individual factors: The need for radiotherapy depends on various factors, including the stage of cancer, the patient's overall health, and the treatment plan recommended by the medical team.

23.What are the side effects of radiotherapy for gastric cancer?
Digestive issues: Diarrhea, nausea, vomiting, loss of appetite
Skin reactions: Redness, dryness, itching, peeling in the treated area
Fatigue: Tiredness and weakness
Mouth sores: Difficulty swallowing or eating
Changes in taste or smell

24.How to evaluate the effectiveness of therapeutic regimens for gastric cancer？
Survival rates: Long-term survival rates of patients treated at the center.
Recurrence rates: How often cancer returns after treatment.
Patient satisfaction: Surveys to assess patient satisfaction with care.
Compliance with national guidelines: Adherence to evidence-based treatment guidelines.
Multidisciplinary team approach: Whether the center has a team of specialists working together (surgeons, oncologists, radiologists, etc.).
Access to advanced technologies: Availability of cutting-edge technologies like robotic surgery, advanced imaging, and immunotherapy.

25.Can gastric cancer patients be cured, and is there a risk of recurrence after cure?

Cure is possible: Early-stage gastric cancer can often be cured with surgery and other treatments.
Recurrence risk: Even after successful treatment, there is a risk of cancer recurrence.
Regular follow-up: Crucial for early detection of any recurrence.
